# Lanthanide(III) Complexes Based on an 18-Membered Macrocycle Containing Acetamide Pendants. Structural Characterization and paraCEST Properties

**DOI:** 10.1021/acs.inorgchem.0c03385

**Published:** 2021-01-20

**Authors:** Goretti Castro, Gaoji Wang, Tanja Gambino, David Esteban-Gómez, Laura Valencia, Goran Angelovski, Carlos Platas-Iglesias, Paulo Pérez-Lourido

**Affiliations:** † Departamento de Química Inorgánica, Facultad de Ciencias, Universidade de Vigo, As Lagoas, Marcosende, 36310 Pontevedra, Spain; § MR Neuroimaging Agents, Max Planck Institute for Biological Cybernetics, 72076 Tübingen, Germany; # Laboratory of Molecular and Cellular Neuroimaging, International Center for Primate Brain Research, Center for Excellence in Brain Science and Intelligence Technology, Chinese Academy of Sciences, 20031 Shanghai, P. R. China; ‡ Centro de Investigacións Científicas Avanzadas and Departamento de Química, Universidade da Coruña, Campus da Zapateira-Rúa da Fraga 10, 15008 A Coruña, Spain

## Abstract

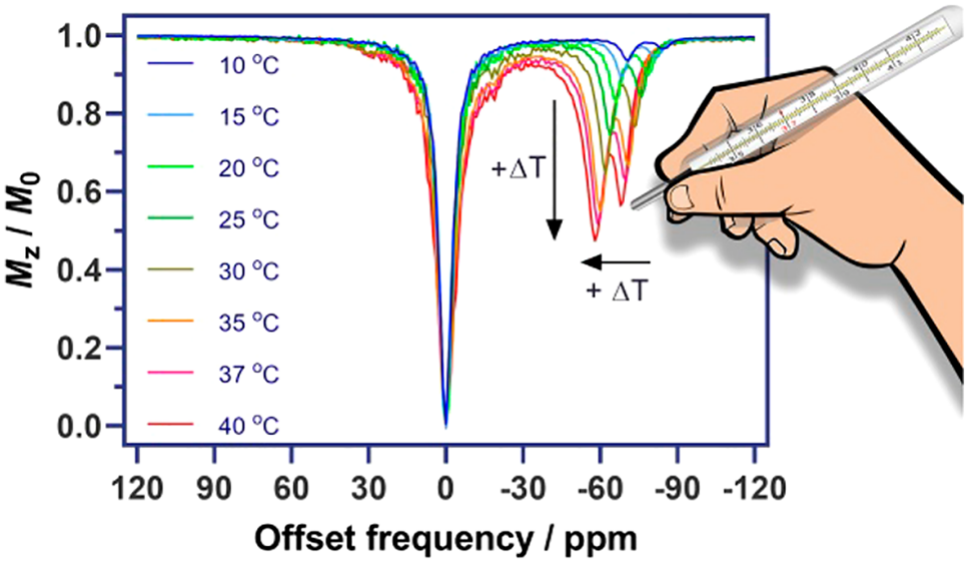

We report a detailed investigation of the coordination properties of macrocyclic lanthanide complexes containing a 3,6,10,13-tetraaza-1,8(2,6)-dipyridinacyclotetradecaphane scaffold functionalized with four acetamide pendant arms. The X-ray structures of the complexes with the large Ln^3+^ ions (La and Sm) display 12- and 10-coordinated metal ions, where the coordination sphere is fulfilled by the six N atoms of the macrocycle, the four O atoms of the acetamide pendants, and a bidentate nitrate anion in the La^3+^ complex. The analogous Yb^3+^ complex presents, however, a 9-coordinated metal ion because one of the acetamide pendant arms remains uncoordinated. ^1^H NMR studies indicate that the 10-coordinated form is present in solution throughout the lanthanide series from La to Tb, while the smaller lanthanides form 9-coordinated species. ^1^H and ^89^Y NMR studies confirm the presence of this structural change because the two species are present in solution. Analysis of the ^1^H chemical shifts observed for the Tb^3+^ complex confirms its *D*_2_ symmetry in aqueous solution and evidences a highly rhombic magnetic susceptibility tensor. The acetamide resonances of the Pr^3+^ and Tb^3+^ complexes provided sizable paraCEST effects, as demonstrated by the corresponding Z-spectra recorded at different temperatures and studies on tube phantoms recorded at 22 °C.

## Abstract 2

A 10-coordinated Tb^3+^ complex shows remarkably a chemical exchange saturation transfer response with a temperature-sensitive chemical shift.

## Introduction

Magnetic resonance imaging (MRI) is a technique commonly used in medical diagnosis that provides three-dimensional images of soft tissues with very high resolution and unlimited depth penetration.^1^ MRI takes advantage of the ^1^H NMR signal of water proton nuclei present in the body, generating contrast due to changes in the density of protons and their longitudinal (*T*_1_) or transverse (*T*_2_) relaxation times.^2^ Both *T*_1_ and *T*_2_ can be shorted in the surrounding of paramagnetic species such as Gd^3+^ or Mn^2+^ chelates, and thus complexes of these metal ions were proposed as contrast agents about 4 decades ago,^3^ subsequently entering clinical practice.^4^

The continuous interest of the MRI community in Gd^3+^-based and, to a lesser extent, Mn^2+^-based contrast agents generated a number of molecular systems with improved properties. This contributed significantly to obtaining new insights in the coordination chemistry of these metal ions in aqueous media.^5,6^ Furthermore, a wide range of the so-called smart or responsive contrast agents were designed to provide a response to different physiologically relevant parameters, such as the pH, temperature, or presence of anions or cations relevant in vivo.^7^ The main drawback of the responsive Gd^3+^ probes is the difficulty of their direct detection: they operate by modifying the NMR signal of water proton nuclei already present in the body, which causes the presence of significant background signals. As a consequence, quantification of the response of Gd^3+^ contrast agents in vivo remains a difficult task because the observed signal depends both on the physiological parameter that induces relaxivity changes and on the probe concentration.^8^

Paramagnetic contrast agents relying on the chemical exchange saturation transfer (paraCEST) approach have attracted great attention during the last 2 decades as alternatives to the Gd- or Mn-based probes.^9^ These agents possess exchangeable protons (typically amide, hydroxyl, or coordinated water molecules) in intermediate-to-slow exchange with bulk water. The paramagnetic shift induced by the metal ion moves the signal of exchangeable protons away from that of bulk water. Consequently, the application of a presaturation pulse at the frequency of the exchanging protons transfers the energy from the saturated spins to the water pool, which decreases its signal intensity.^10^ The relatively large chemical shift difference between the signals of exchangeable protons and bulk water (Δω), induced by the paramagnetic ion, implies that the proton exchange rate (*k*_ex_) can be faster, while still maintaining the slow-to-intermediate exchange regime (*k*_ex_ ≤ Δω) to observe the CEST effect.^11^ Moreover, the paramagnetic shift can, in some cases, cause the differentiation of two nonequivalent exchangeable protons to result in two separate CEST signals. This feature is beneficial because it allows for ratiometric analyses and thus possibly quantitative estimation of the physiological parameters.^12^

**Chart 1:**
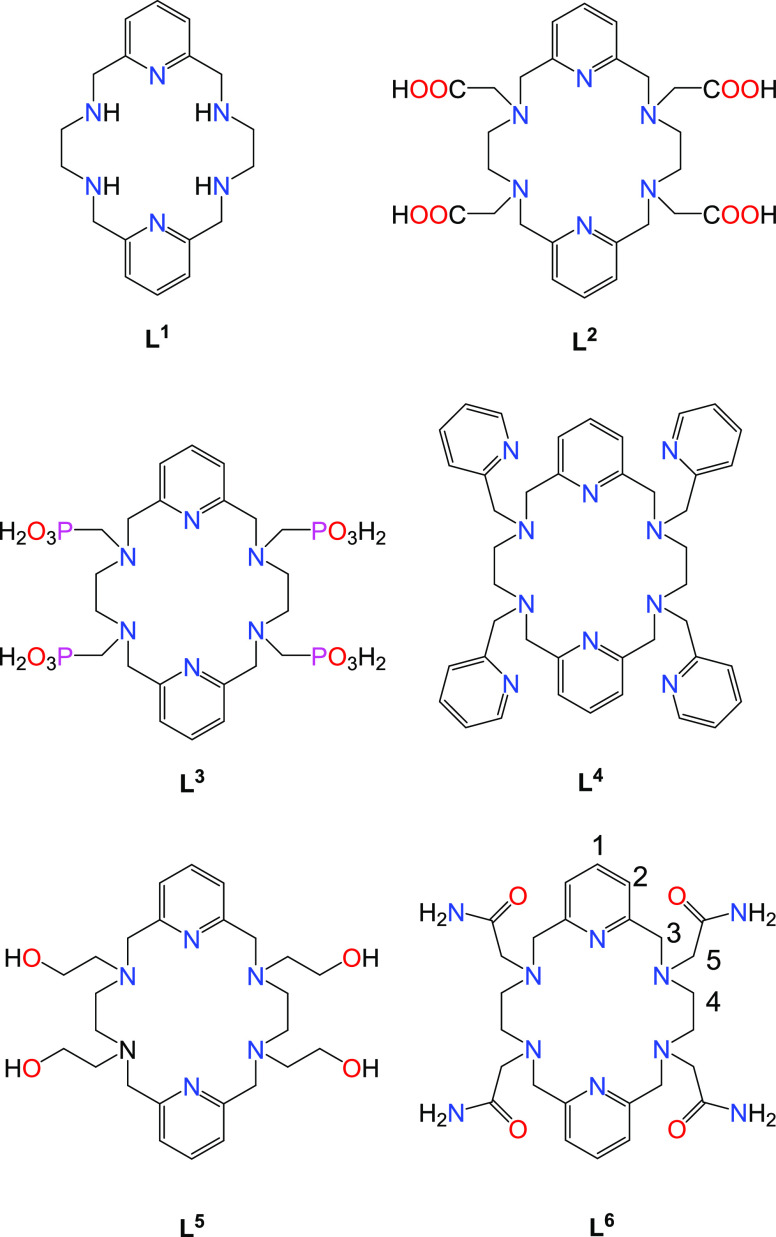
Ligands Discussed in the Present Work

Because of such an advantageous prospective, complexes of both paramagnetic lanthanide and transition-metal ions were extensively investigated as potential paraCEST candidates.^13−15^ In the particular case of lanthanide ions, most of the complexes investigated in this context were derivatives of cyclen because this type of chelator often forms very stable and inert complexes when functionalized with four pendant arms.^16^ However, azamacrocyclic platforms other than cyclen were also investigated as possible chelators to form inert complexes with affirmative CEST features. For instance, the 18-membered macrocyclic ligand L^1^ (Chart 1) was functionalized with different pendant arms to accommodate the high coordination numbers usually observed for the Ln^3+^ ions in solution. Along these lines, the derivatives containing acetate^17^ and methylenephosphonic acid^18^ groups (L^2^ and L^3^, respectively) were prepared and reported more than 15 years ago. Moreover, the same macrocyclic platform was also functionalized with neutral pyridyl^19^ and hydroxyethyl^20,21^ pendant arms (L^4^ and L^5^). This family of ligands generally provides 10-coordinated complexes, although in some cases, decoordination of one of the pendant arms is observed along the second half of the lanthanide series. Interestingly, the [LnL^5^]^3+^ complexes containing hydroxyl groups were found to be exceptionally inert with respect to complex dissociation, remaining intact in a 1 M HCl solution over periods of months. Finally, in a recent work, we reported the derivative L^6^ containing acetamide pendants and demonstrated that its Eu^3+^ complex displays high kinetic inertness and provides a strong pH-sensitive CEST effect due to the amide protons.^22^

Following these encouraging results, we performed further studies with this promising chelator. Herein we report a detailed investigation of the structure of several lanthanide complexes of L^6^, both in the solid state and in solution. The X-ray structures of three complexes are presented (La^3+^, Sm^3+^, and Yb^3+^). The structure in solution was assessed through a detailed study of the ^1^H and ^89^Y NMR spectra, including a detailed analysis of the paramagnetic shifts observed for the Tb^3+^ derivative. Finally, the paraCEST spectra of the Pr^3+^ and Tb^3+^ complexes recorded at different temperatures are presented and analyzed quantitatively using the Bloch–McConnell (BM) theory to determine the exchange rates of amide protons.

## Results and Discussion

### Synthesis and Characterization of the Ligand and Metal Complexes

Ligand L^6^ was obtained by alkylation of the 3,6,10,13-tetraaza-1,8(2,6)-dipyridinacyclotetradecaphane precursor (L^1^)^23^ with bromoacetamide, as described previously.^22^ Compounds with the formula [LnL^6^](NO_3_)_3_·*x*H_2_O (La–Lu, except Pm and Eu, *x* = 2–4) were isolated with good yields (59–86%) after direct reaction in methanol of L^6^ with the corresponding hydrated lanthanide nitrate. The peaks due to the [Ln(L^6^-H)(NO_3_)]^+^ and [Ln(L^6^-2H)]^+^ entities observed in the mass spectrometry (MS) spectra (positive-mode electrospray ionization, ESI^+^) confirm formation of the complexes.

### X-ray Crystal Structures

Crystals of [LaL^6^(NO_3_)]_2_[La(NO_3_)_6_]·NO_3_·4CH_3_OH were obtained by the slow evaporation of a methanolic solution of the ligand containing an excess of La(NO_3_)_3_. This compound crystallizes in the centrosymmetric *C*2/*c* monoclinic space group, and the asymmetric unit encompasses the [LaL^6^(NO_3_)]^2+^ complex, half of a [La(NO_3_)_6_]^3–^ anion, half of a nitrate anion, and two methanol molecules. The [La(NO_3_)_6_]^3–^ entity (Figure S1) was previously found in crystals of different cationic La^3+^ complexes, presumably aiding crystallization because of its large size.^24^

**Figure 1:**
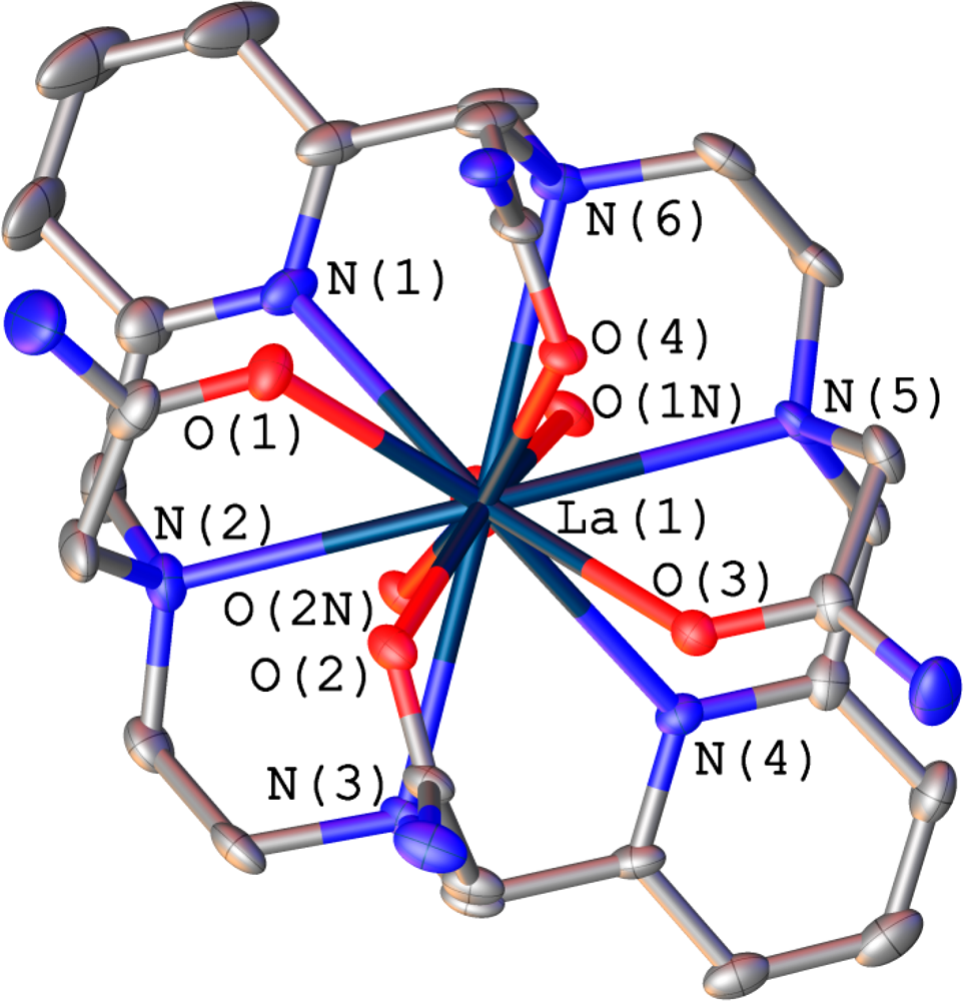
Structure of the [LaL^6^(NO_3_)]^2+^ cation present in crystals of [LaL^6^(NO_3_)]_2_[La(NO_3_)_6_]·NO_3_·4CH_3_OH. H atoms are omitted for simplicity. The ORTEP plot is at the 30% probability level.

The [LaL^6^(NO_3_)]^2+^ cation shows that the La^3+^ ion is coordinated by the six N atoms of the macrocycle skeleton, the four O atoms from the amide groups, and two of the O atoms of a nitrate group acting as a bidentate ligand, which results in coordination number 12. The bond distances of the metal coordination environment are collected in Table 1, while a view of the structure of the complex is presented in Figure 1. The coordination polyhedron around the La^3+^ ion can best be described as a twisted icosahedron (Figure S2), as indicated by the analysis performed with the *SHAPE* program.^25,26^ The nitrate anion in [LaL^6^(NO_3_)]^2+^ provides a slightly asymmetric bidentate coordination, with La–O distances similar to those reported for other 12-coordinated La^3+^ complexes containing bidentate ligands.^27^

**Table 1:** Bond Distances (Å) of the Metal-Coordination Spheres Obtained for the [LnL^6^]^3+^ Complexes with X-ray Diffraction Measurements

	La	Sm	Yb
Ln(1)–N(1)	2.865(7)	2.589(8)	2.4787(16)
Ln(1)–N(2)	2.919(7)	2.641(8)	2.6436(17)
Ln(1)–N(3)	2.841(6)	2.672(8)	2.5790(17)
Ln(1)–N(4)	2.826(6)	2.572(7)	2.4831(16)
Ln(1)–N(5)	2.876(6)	2.659(8)	2.6278(16)
Ln(1)–N(6)	2.887(6)	2.669(8)	2.5467(16)
Ln(1)–O(1)	2.569(6)	2.511(7)	2.3135(14)
Ln(1)–O(2)	2.622(5)	2.487(8)	2.2704(14)
Ln(1)–O(3)	2.611(5)	2.573(8)	
Ln(1)–O(4)	2.654(5)	2.535(7)	2.2746(14)
Ln(1)–O(1N)	2.733(6)		
Ln(1)–O(2N)	2.710(5)		

The [LaL^6^(NO_3_)]^2+^ complex presents an unprecedented conformation of the ligand in which the four pendant arms of L^6^ are oriented to the same side of the macrocyclic unit, giving a syn conformation. The macrocyclic unit is not folded or twisted but shows a plateau conformation. The four amine N atoms define a least-squares plane [root-mean-square (rms) = 0.238 Å] that contains the La^3+^ ion, while the pyridine N atoms are ca. 1.07 Å below that plane. As a result, the N(4)–La(1)–N(1) angle [125.17(19)°] is not linear. The pyridyl units are slightly tilted with respect to each other, with the least-squares planes intersecting at 28.2°. The two chelate rings associated with binding of the ethylenediamine units adopt the same conformation, which can be defined as δδ or λλ.^28^ The layout of the four acetamide pendant groups provides the second source of chirality, leading to the presence of (centrosymmetrically related) Λ(λλ) or Δ(δδ) enantiomers in the crystal lattice.^29^

**Figure 2:**
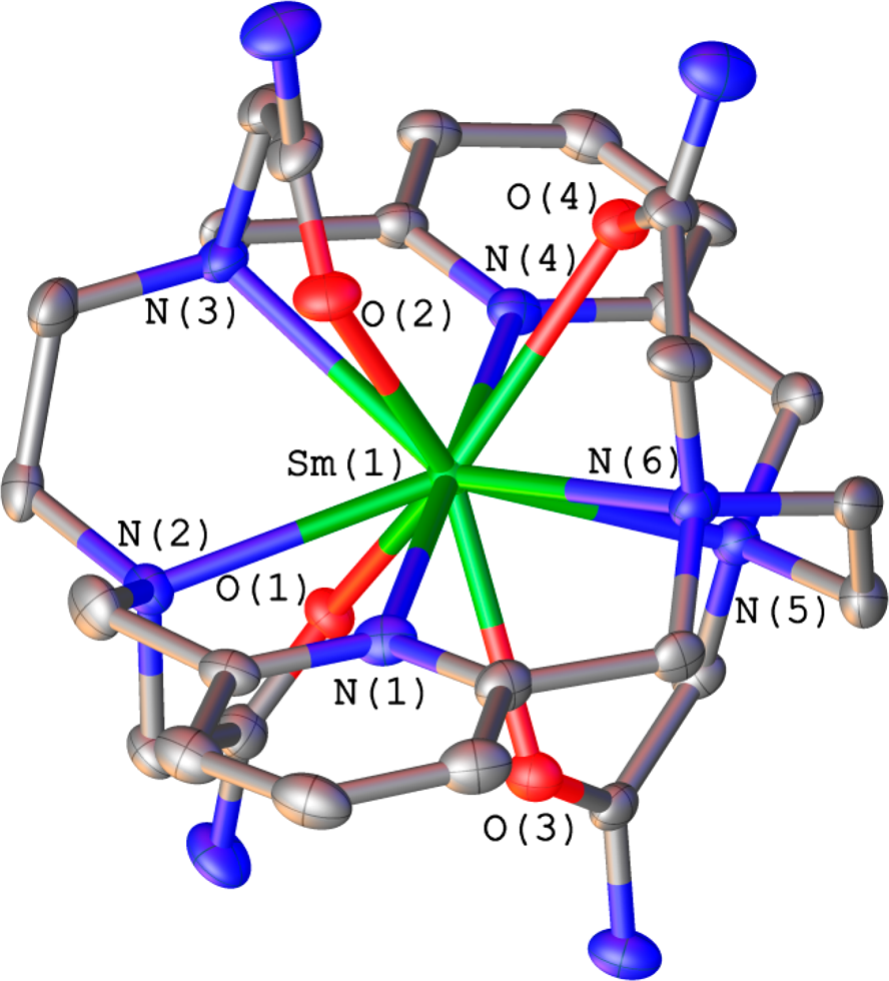
Structure of the [SmL^6^]^3+^ cation present in crystals of [SmL^6^](NO_3_)_2.91_·Br_0.09_. H atoms are omitted for simplicity. The ORTEP plot is at the 30% probability level.

Crystals of the formula [SmL^6^](NO_3_)_2.91_·Br_0.09_ and [YbL^6^](NO_3_)_2.7_·Br_0.3_·3H_2_O were obtained by the slow evaporation of an aqueous solution of the complex. The small fraction of bromide anions present in these crystals is likely due to the presence of KBr impurities in the batch of ligands used for preparation of the complex. Sm^3+^ crystals also contain the [SmL^6^]^3+^ cation (Figure 2) and nitrate anions. This compound crystallizes in the noncentrosymmetric monoclinic *C*_2_ space group, and the asymmetric units contain one and two halves of the [LnL^6^]^3+^ units, six nitrate anions, and water molecules.

The [SmL^6^]^3+^ cation contains a 10-coordinated metal ion that is directly coordinated by the six donor atoms of the macrocyclic fragment and the four O atoms of the acetamide pendants. The overall structure is very similar to that reported previously for the Eu^3+^ and Y^3+^ derivatives.^22^ The ligand adopts a *twist-wrap* conformation characterized by a nearly linear N(1)–Sm(1)–N(4) angle [179.3(4)°].^17^ The pyridyl rings are twisted with respect to the N(1)–Sm(1)–N(4) axis, with their least-squares planes intersecting at 18.2°. The (5-membered) chelate rings generated by coordination of the ethylenediamine groups adopt identical conformations [(δδ) or (λλ)]. The coordination polyhedron can best be defined as a sphenocorona (Figure S3),^30^ where the quadrangular faces are defined by N(1), N(2), O(1), and O(3) (rms = 0.234 Å) and O(1), O(3), N(4), and N(5) (rms = 0.228 Å).

**Figure 3:**
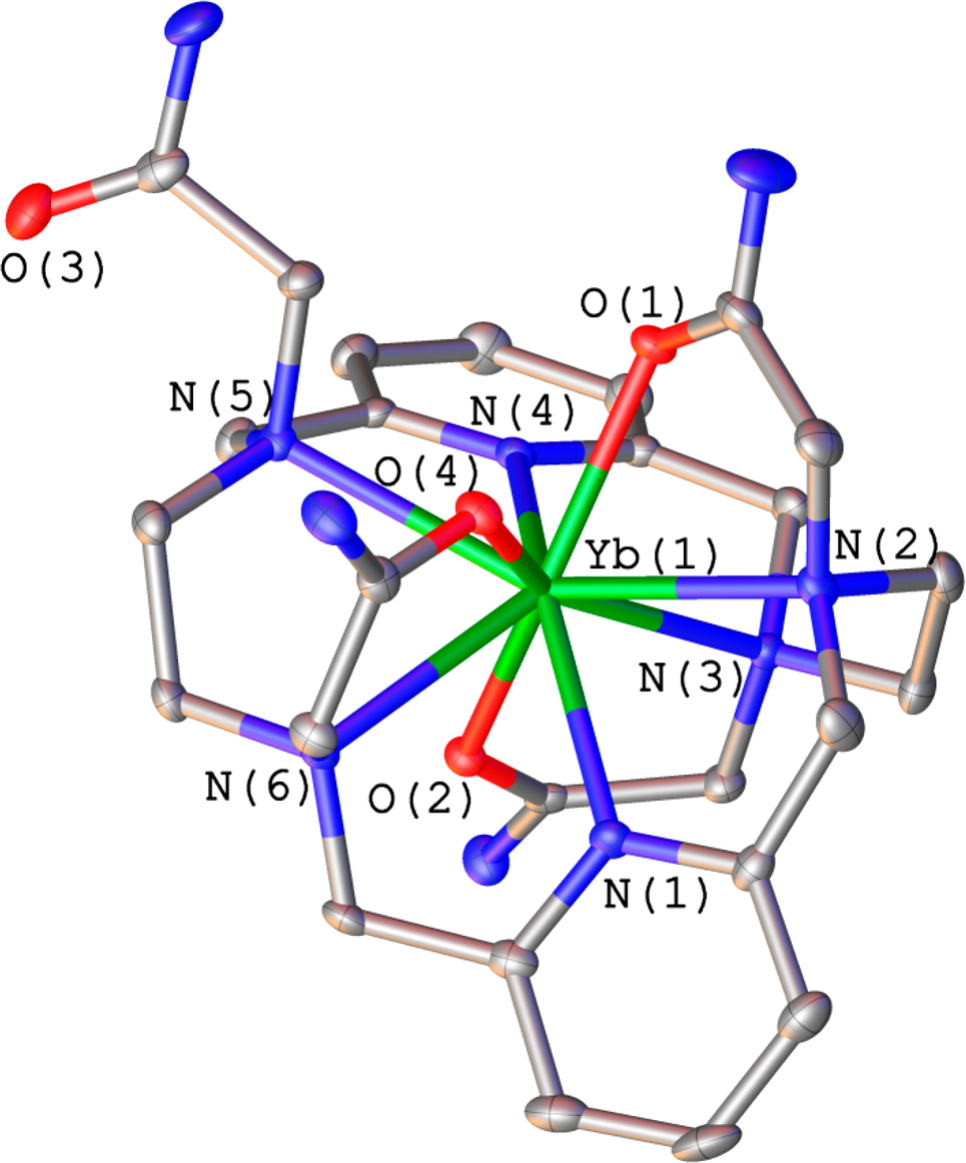
Structure of the [YbL^6^]^3+^ cation present in crystals of [YbL^6^](NO_3_)_2.7_·Br_0.3_·3H_2_O. H atoms are omitted for simplicity. The ORTEP plot is at the 30% probability level.

Crystals of the Yb^3+^ compound contain the [YbL^6^]^3+^ complex, in which the metal ion is 9-coordinated by the ligand (Figure 3). One of the acetamide pendant arms remains uncoordinated, with the ligand adopting a *twist-fold* conformation characterized by a N(1)–Yb(1)–N(4) angle of 144.04(5)° and a dihedral angle between the pyridine units of 72.4(1)°. A similar structure was found for complexes formed by the carboxylate analogue L^2^ and the heaviest lanthanide ions.^17^ Changes in the coordination number from 10 to 9 across the lanthanide series coming as a result of lanthanide contraction are fairly common.^31^ The coordination geometry is close to a spherical tricapped trigonal prism (Figure S4). The upper tripod of the polyhedron is defined by O(1), O(4), and N(5), while the lower tripod contains N(3), N(1), and O(2). These two triangular faces are almost parallel, intersecting at 3.6°. The capping tripod is occupied by N(2), N(4), and N(6).

The syn structure of the La^3+^ complex is characterized by Ln–N distances involving the pyridyl donor atoms [N(1) and N(4)] comparable to those with amine N atoms. However, the Ln–N(1) and Ln–N(4) bonds are significantly shorter than those involving amine N atoms in [SmL^6^]^3+^ and [YbL^6^]^3+^, a situation that is commonly observed when the pendant arms coordinate from both sides of the macrocyclic mean plane.^17−22^ The Sm–O and Sm–N distances observed for the [SmL^6^]^3+^ complex are very similar to those observed for the carboxylate analogue [SmHL^2^].^17^ The Yb–O distances in [YbL^6^]^3+^ are in the normal range observed for 9-coordinated Yb^3+^ complexes containing amide pendant groups.^32^

### Structures of the Complexes in Solution

**Figure 4:**
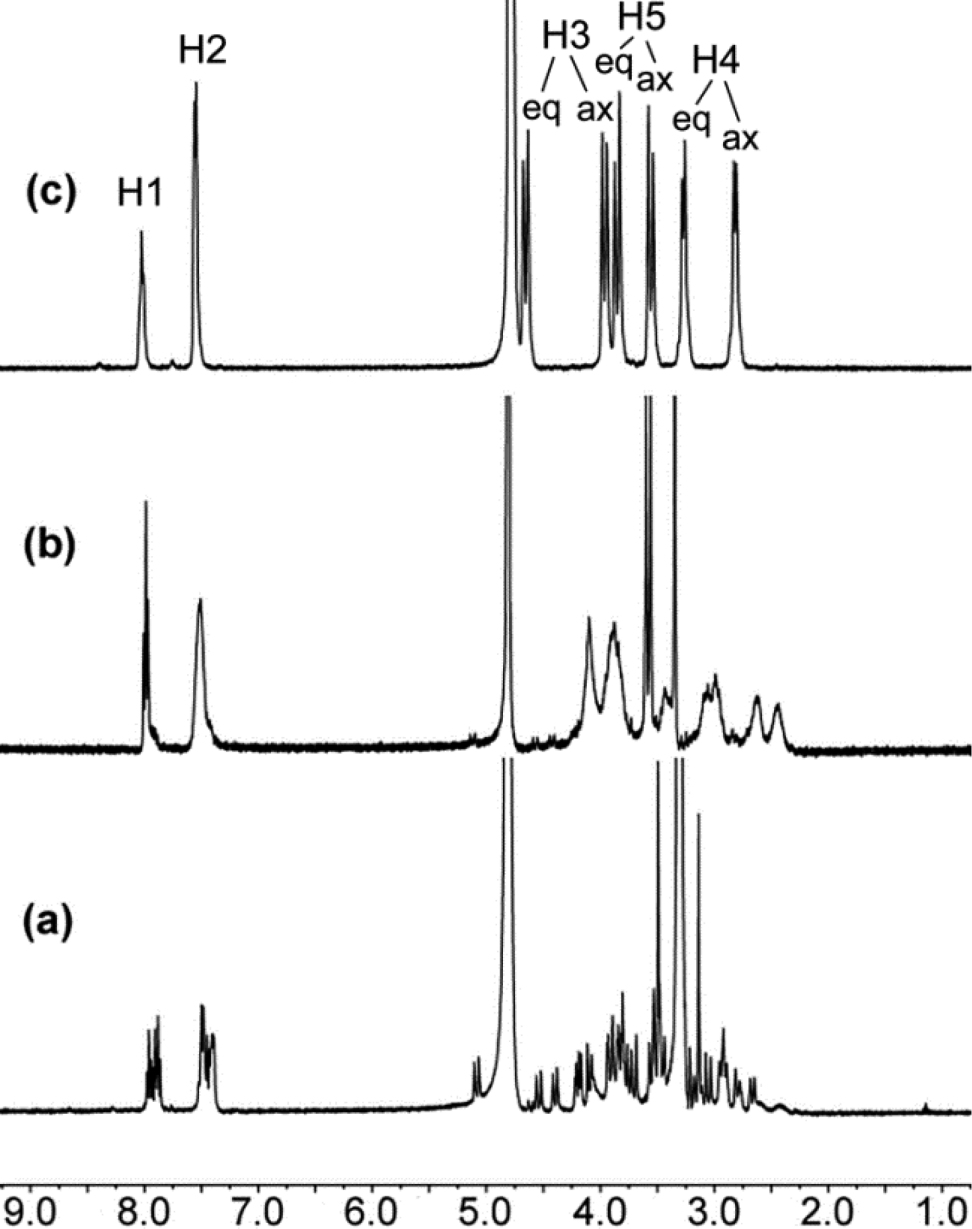
^1^H NMR spectra of [LaL^6^]^3+^ (D_2_O, 400 MHz, pH 7.0, 25 °C): (a) immediately after dissolution of the complex; (b) after 3 days at RT; (c) after heating of the solution at 80 °C for 10 min.

The ^1^H NMR spectrum of diamagnetic [LaL^6^]^3+^ recorded in a D_2_O solution is rather complex, which indicates the presence of two species in solution (Figure 4, spectrum a). The spectrum changes slowly and irreversibly with time, a process that speeds up upon heating of the solution. The spectrum obtained after heating is well-resolved, showing the eight signals expected for a *D*_2_ symmetry in solution. We attribute this behavior to the formation of a kinetic species with a syn conformation of the macrocycle, as observed in the X-ray structure (*C*_2_ symmetry), which evolves to the thermodynamically stable species with *D*_2_ symmetry in solution. The spectrum of the Ce^3+^ complex is poorly defined, likely for the same reason.

**Figure 5:**
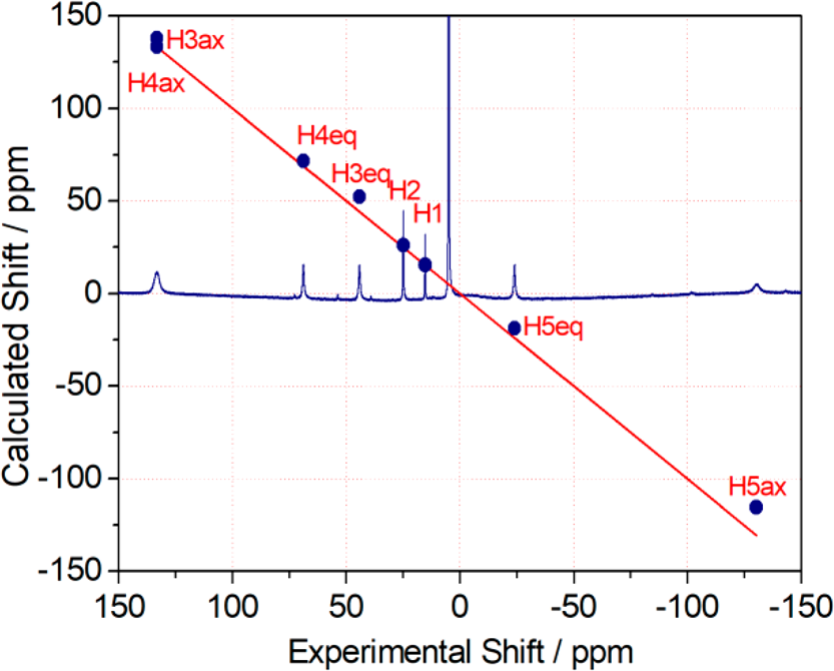
^1^H NMR spectrum of the [TbL^6^]^3+^ complex recorded in a D_2_O solution (400 MHz, pH 7.0, 25 °C) and plot of the experimental ^1^H NMR shifts versus those calculated from the contact and pseudocontact contributions (see the text). The red line is the identity line.

The complexes with Pr^3+^, Nd^3+^, Sm^3+^, Eu^3+^, and Tb^3+^ present well-resolved spectra with eight paramagnetically shifted resonances, which points to a *D*_2_ symmetry of the complexes in solution (Figures 5 and S5–S8). These results are in full agreement with the X-ray structures described above. The ^1^H NMR spectra of the [LnL^6^]^3+^ complexes (Ln = Pr–Tb, except Pm) were assigned on the basis of COSY spectra and line-width analysis (Table 2). The observed chemical shifts are similar to those previously reported for the [LnL^5^]^3+^ complexes,^21^ indicating similar magnetic anisotropies and thus similar solution structures.^33^

**Table 2:** ^1^H NMR Shifts (D_2_O, 25 °C, pH 7.0, 400 MHz) Observed for [LnL^6^]^3+^ Complexes^a^

	H1	H2	H3ax	H3eq	H4ax	H4eq	H5ax	H5eq
La^b^	8.04	7.58	3.98	4.68	2.84	3.30	3.58	3.87
Ce	9.92	8.64	14.10	9.92	15.26	13.89	–8.08	2.25
Pr	9.40	10.28	20.56	15.53	22.32	23.48	–15.20	4.08
Nd	13.83	15.22	14.45	15.22	1.49	12.22	–6.64	1.19
Sm^c^	8.36	8.07	7.00	4.82	4.43	3.15	1.52	2.73
Eu	2.45	–0.77	–9.43	–15.05	–1.62	–19.13	18.50	0.72
Tb	15.20	24.79	133.14	44.08	133.14	68.63	–130.44	–24.14

a See Chart 1 for labeling.

b ^3^*J*_1,2_ = ^3^*J*_2,1_ = 7.9 Hz; ^2^*J*_3ax,3eq_ = ^2^*J*_3eq,3ax_ = 15.9 Hz; ^3^*J*_4ax,4eq_ = ^3^*J*_4eq,4ax_ = 10.0 Hz; ^2^*J*_5ax,5eq_ = ^2^*J*_5eq,5ax_ = 16.6 Hz.

c ^3^*J*_1,2_ = ^3^*J*_2,1_ = 7.7 Hz; ^2^*J*_3ax,3eq_ = ^2^*J*_3eq,3ax_ = 15.8 Hz; ^3^*J*_4ax,4eq_ = ^3^*J*_4eq,4ax_ = 10.2 Hz; ^2^*J*_5ax,5eq_ = ^2^*J*_5eq,5ax_ = 16.1 Hz.

The structure in solution of the [TbL^6^]^3+^ complex was further investigated by analyzing the observed ^1^H NMR shifts, which are the result of diamagnetic (δ^dia^) and paramagnetic (δ^para^) contributions (eq 1).
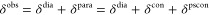
1

The diamagnetic contributions to the observed shifts were obtained from the chemical shifts observed for the diamagnetic [LaL^6^]^3+^ analogue (Table 2). The paramagnetic shifts induced by Tb^3+^ are the result of both the contact (δ^con^) and pseudocontact (δ^pscon^) mechanisms,^34^ but only the latter encodes information on the position of the observed nuclei with respect to the paramagnetic center. Thus, we estimated the contact contributions by calculating the hyperfine coupling constants *A*/*ℏ* responsible for the contact shifts using density functional theory (DFT). Contact shifts are directly proportional to the hyperfine coupling constant at the observed nuclei, as given by eq 2, where ⟨*S*_*z*_⟩ is the spin expectation value of the lanthanide ion,^35^ γ_I_ is the nuclear gyromagnetic ratio, *k* is the Boltzmann constant, and β is the Bohr magneton.^36^
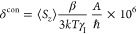
2

The values of *A*/ℏ were estimated using calculations on the [GdL^6^]^3+^ analogue, following the methodology reported previously.^37^ DFT provides a 10-coordinated structure for [GdL^6^]^3+^ very similar to those observed in the solid state for the Sm^3+^, Eu^3+^, and Y^3+^ derivatives (Figure S9). Contact shifts were subsequently obtained by using ⟨*S*_*z*_⟩ = 22.0.^21^

Separation of the contact and pseudocontact shifts shows that the paramagnetic shifts are generally dominated by the pseudocontact mechanism, with the contact shift being more important for equatorial protons (Table 3). This has been attributed to distinct Ln–N–C–H dihedral angles characterizing the axial (∼80°) and equatorial (∼175°) protons because contact shifts show a Karplus-like behavior on this angle.^37^

**Table 3:** Paramagnetic ^1^H NMR Shifts (δ^para^, D_2_O, 25 °C, pH 7.0, 400 MHz), Hyperfine Coupling Constants (*A*/ℏ), and Pseudocontact and Contact and Contributions Obtained for the [TbL^6^]^3+^ Complex^a^

	H1	H2	H3ax	H3eq	H4ax	H4eq	H5ax	H5eq
δ^para^	7.16	17.21	129.16	39.4	130.3	65.33	–134.02	–28.01
*A*/*ℏ*/10^6^ rad s^–1^ b	–0.0271	–0.0253	0.07848	–0.3145	0.00000	–0.5870	0.10267	–0.25510
δ^con^	–1.67	–1.56	4.85	–19.43	0.00	–36.27	6.34	–15.76
δ^pscon^	8.83	18.77	124.31	58.83	130.3	101.6	–140.36	–12.25

a See Chart 1 for labeling.

b Calculated for [GdL^6^]^3+^ at the TPSSh/SCRECP/EPR-III level (see the Computational Details).

The pseudocontact contribution can be expressed as in eq 3 if the reference frame coincides with the main directions of the magnetic susceptibility tensor **χ**,^38^ where  and *x*, *y*, and *z* are the Cartesian coordinates of the observed nucleus relative to the position of a Ln^3+^ ion placed at the origin, while Δχ_ax_ and Δχ_rh_ are the axial and rhombic components of the diagonalized magnetic susceptibility tensor.
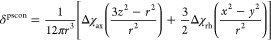
3

A comparison of the experimental and calculated pseudocontact shifts, obtained with the geometry of [TbL^6^]^3+^ optimized by means of DFT, is presented in Figure 5 (see also Table S1). The excellent agreement between the two sets of data unambiguously establishes that the [TbL^6^]^3+^ complex presents a structure in solution very similar to that observed in the solid state for the Sm^3+^ and Eu^3+^ analogues (see also Figure 5).

The best fit of the data provided a highly rhombic susceptibility tensor characterized by Δχ_ax_ = −20.5 ± 0.5 × 10^–32^ and Δχ_rh_ = −19.9 ± 1.1 × 10^–32^ m^3^. These values are very similar to those determined previously for [TbL^5^]^3+^, confirming that the two complexes present similar structures in solution.

The ^1^H NMR spectra of the complexes with the heaviest Ln^3+^ ions (Dy–Lu) are complicated likely because of the fact that one of the pendant arms of the ligand is not coordinated to the Ln^3+^ ion, which is in line with the X-ray structure of the Yb^3+^ complex. The spectrum of the Yb^3+^ complex is well-defined, showing 30 signals expected for a 9-fold coordination of the ligand in the range ∼151 to −70 ppm (Figure S10).

**Figure 6:**
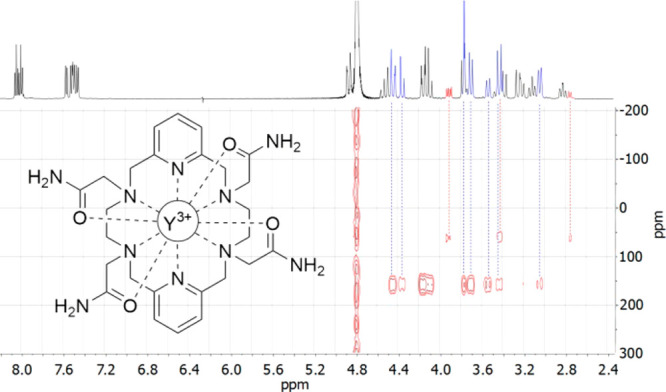
^1^H–^89^Y HMQC NMR spectrum of [YL^6^]^3+^ (D_2_O, 500 MHz, pH 7.0, 25 °C). The signals of the major (9-coordinated) and minor (10-coordinated) species present in solution are highlighted in blue and red, respectively.

The ^1^H NMR spectrum of the [YL^6^]^3+^ complex displays two sets of signals, one due to a symmetrical *D*_2_ species and a second one with higher intensity due to a complex species having *C*_1_ symmetry (Figure 6). The ionic radius of Y^3+^ (1.075 Å for coordination number 9) is very similar to those of the heaviest Ln^3+^ ions (1.072 Å for Ho^3+^ with coordination number 9).^39^ To investigate in more detail the nature of the species present in solution, we sought to apply an empirical correlation developed recently, which relates the observed ^89^Y NMR shifts and the types of donor atoms coordinated in solution.^40^ Thus, we measured the ^89^Y NMR shifts in an attempt to confirm the presence in solution of both the 10- and 9-coordinated species suggested by the crystallographic study. The ^89^Y NMR shifts were obtained by using ^1^H–^89^Y HSQC NMR experiments, which allow a fast acquisition by overcoming the very long relaxation times of the ^89^Y nucleus.^41^ The ^1^H–^89^Y HSQC NMR spectrum presents cross-peaks relating the signals of the major species in solution and an ^89^Y NMR signal at 157 ppm, while for the minor species present in solution, the ^89^Y NMR signal was observed at 51 ppm (Figure 6). These chemical shifts are in excellent agreement with those calculated using the relationship shown in eq 4, where *S*_N_am__, *S*_N_py__, and *S*_O_a__ are the shielding contributions of amine, pyridine, and amide donor atoms (68.1, 85.7, and 89.5 ppm, respectively) and *n*_N_am__, *n*_N_py__, and *n*_O_a__ are the number of donor atoms of each type:^40^
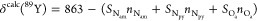
4

Using *n*_N_am__ = 4, *n*_N_py__ = 2, and *n*_O_a__ = 4 gives a calculated ^89^Y chemical shift of 61.2 ppm, which is in very good agreement with the experimental value of 51 ppm. An identical analysis but using *n*_O_a__ = 3 gives a calculated value of 150.7 ppm, again in excellent agreement with the experimental value observed for the major (unsymmetrical) species present in solution (157 ppm). Thus, these results confirm the presence in solution of 9-coordinated species in the case of the smallest Ln^3+^ ions and Y^3+^, with a smaller population of the 10-coordinated species having *D*_2_ symmetry.

The ^89^Y shielding constants of the 10- and 9-coordinated forms of [YL^6^]^3+^ were calculated using DFT (see the Computational Details). The chemical shielding values obtained from these calculations are compiled in Table 4. The subsequent calculation of ^89^Y chemical shifts requires determination of the shielding constant of a reference, generally [Y(H_2_O)_8_]^3+^ (0.0 ppm).^42^ The shielding constants calculated for the 9- and 10-coordinated forms of [YL^6^]^3+^ differ by 105.7 ppm, in nice agreement with the experimental value of Δδ = 106 ppm. This value is close to the empirical contribution of an amide donor of *S*_O_a__ = 89.5 ppm.^40^ However, the use of the shielding constant of [Y(H_2_O)_8_]^3+^ provided ^89^Y NMR chemical shifts with large deviations from the experimental value. We recently showed that the agreement between the experimental and calculated ^71^Ga chemical shifts improved significantly upon inclusion of an explicit second solvation shell.^43^ We thus performed calculations on the [Y(H_2_O)_8_]^3+^·16H_2_O system, which includes 16 water molecules in the second sphere involved in hydrogen bonds with the coordinated water molecules (Figure S11). The structure calculated for [Y(H_2_O)_8_]^3+^ presents the expected square-antiprismatic coordination environment with Y–O distances in the range of 2.360–2.371 Å, in good agreement with the distances observed in the solid state (2.31–2.38 Å).^44^ Inclusion of the second solvation shell broadens the range of Y–O distances (2.35–2.47 Å). The second solvation shell significantly affects the isotropic shielding constant, which decreases by ∼64 ppm. The ^89^Y NMR chemical shifts of [Y(H_2_O)_8_]^3+^ calculated using [Y(H_2_O)_8_]^3+^·16H_2_O as a reference (Table 4) are in reasonably good agreement with the experimental values of 157 and 51 ppm. σ_iso_ can be separated into the usual diamagnetic (σ_d_) and paramagnetic (σ_p_) contributions (Table 4).^45^ The results show that the distinct chemical shifts are mainly related to variations in the σ_p_ values, which originate from the ability of the applied field to mix excited states into the ground state. The σ_d_ values calculated for all systems are, however, very similar, as would be expected.

**Table 4:** Isotropic ^89^Y Shielding Constants (σ_iso_), Paramagnetic (σ_p_) and Diamagnetic Contributions (σ_d_), and ^89^Y Chemical Shifts Calculated with DFT^a^

	σ_iso_	σ_d_	σ_p_	δ_calc_
[Y(L^6^)]^3+^ (CN = 10)	2568.8	3783.4	–1214.6	180.8
[Y(L^6^)]^3+^ (CN = 9)	2674.5	3777.6	–1103.1	75.1
[Y(H_2_O)_6_]^3+^	2813.9	3761.6	–947.7	
[Y(H_2_O)_6_]^3+^·16H_2_O	2749.6	3771.7	–1022.1	0

a DFT calculations using the GIAO method in aqueous solution (PCM) at the TPSSh/DKH2/Def2-TZVPP level.

### CEST Experiments

The crystal structures of the Pr^3+^ and Tb^3+^ complexes revealed that the complexes are lacking coordinated water molecules. However, they possess exchangeable amide proton pools suitable for exploitation of the CEST effect. Thus, to investigate the potential application of these complexes as paraCEST agents, we conducted a series of NMR and MRI studies. Both solutions of [TbL^6^]^3+^ and [PrL^6^]^3+^ showed two notable paraCEST signals at different temperatures. For the Pr^3+^ complex, two CEST peaks at −2.8 and −8.0 ppm are strong but close to the peak of water protons at 25 °C (Figure S12). For the Tb^3+^ complex, we performed a set of experiments with the temperature ranging from 10 to 40 °C. For each temperature, we recorded the Z-spectra using seven different saturation powers from 2.5 to 30 μT (see the Experimental Section). The results allowed us to follow the position (shift) of the CEST peaks, as well as to calculate the exchange rates of these two exchangeable protons as a function of the temperature.

**Figure 7:**
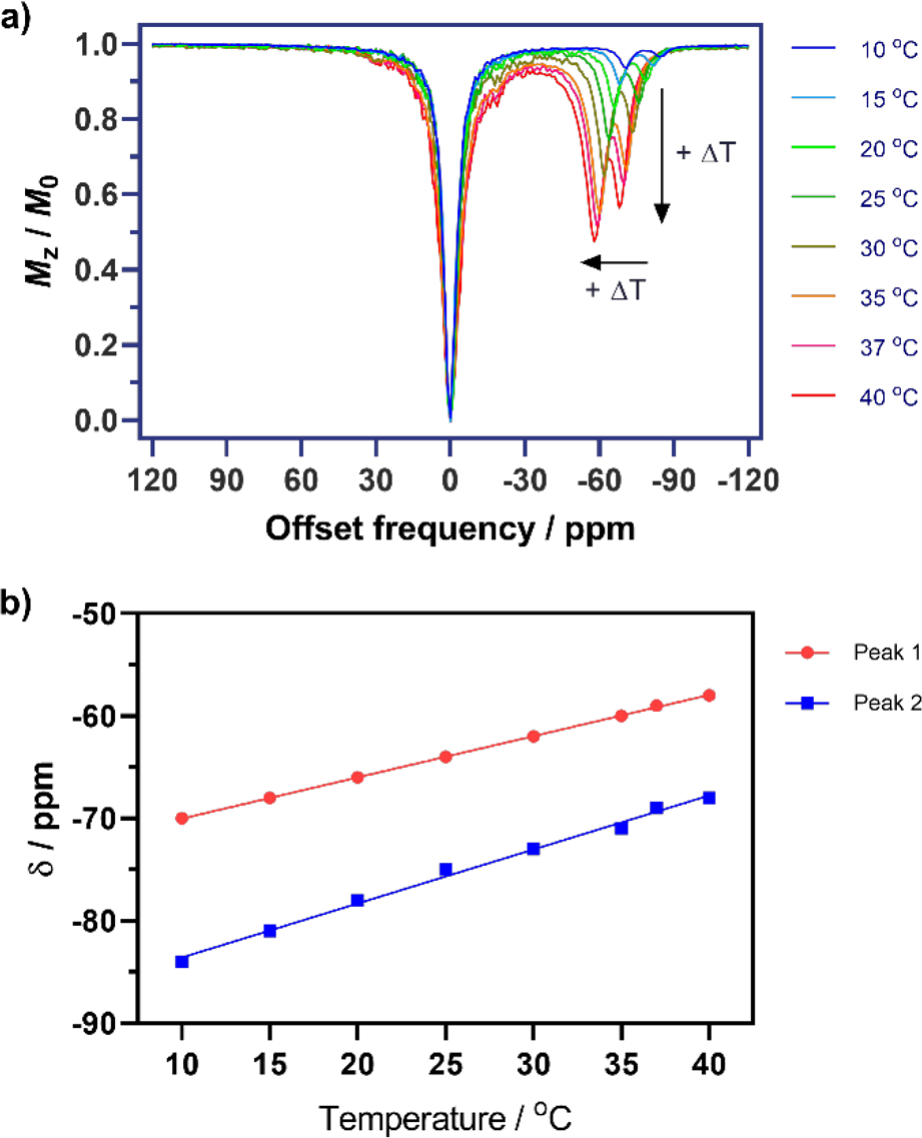
Top: CEST spectra recorded at 7 T of a solution containing [TbL^6^]^3+^ (10 mM in 25 mM PBS, pH 7.0) at different temperatures using a saturation time of 8 s and a saturation power of *B*_1_ = 20 μT. Bottom: Variation of the chemical shifts of amide protons with temperature.

The CEST signals of [TbL^6^]^3+^ are highly paramagnetically shifted and strongly dependent on the temperature: these two peaks shift from −64 and −76 ppm to −60 and −70 ppm from 25 to 37 °C, respectively (Figure 7). Over the entire temperature range examined, the sensitivity of the chemical shift to temperature was 0.54 or 0.41 ppm/°C for the low or high field signal, respectively (Figure 7). This is an interesting result because this particular feature of [TbL^6^]^3+^ can be potentially exploited for measuring the temperature distribution in a living subject: here both signals can be used to determine the temperature while concurrently acting as controls for the other peak; i.e., the distance between these two peaks is also temperature-dependent. The temperature coefficients determined for [TbL^6^]^3+^ are similar to those reported for ^1^H resonances of paramagnetic cobalt(II) and iron(II) complexes suggested for registration of the temperature.^46^

The exchange rates of the CEST-active protons, *k*_ex_, were determined using the previously established qCEST method,^47^ by fitting a series of multi-*B*_1_ Z-spectra according to the BM equations. As expected, the *k*_ex_ values are quite low at lower temperatures and less sensitive compared to those in the higher-temperature region. The *k*_ex_ values are as low as <100 Hz at 10 °C, increasing to up to 3 kHz at 40 °C (Table S2). Of the two peaks, the one with lower shift always exchanges faster. The *k*_ex_ values determined at different temperatures for [TbL^6^]^3+^ were used to estimate the activation parameters for amide proton exchange using the Eyring equation (Figure S14). The most shifted signal is characterized by Δ*H*^⧧^ = 45.3 ± 2.6 kJ mol^–1^ and Δ*S*^⧧^ = −38.4 ± 2.0 J mol^–1^ K^–1^. The signal with a smaller shift (and fast exchange) yields Δ*H*^⧧^ = 47.4 ± 2.2 kJ mol^–1^ and Δ*S*^⧧^ = −27.5 ± 1.2 J mol^–1^ K^–1^. The enhanced exchange rate of the latter amide protons appears to be related to a substantially lower entropy barrier, presumably due to the formation of a more favored (i.e., less ordered) transition state, which likely possesses a more extensive dispersion of charge.^48^

Importantly, the *k*_ex_ values at 37 °C are in the optimal range of 1.5–2.5 kHz, which combined with the high number of the NMR equivalent and shifted protons (two groups, each with four protons) makes this platform very attractive for the further development of potent paraCEST probes.^15,22^ Concurrently, the obtained exchange rates for [PrL^6^]^3+^ were in a range similar to that for [TbL^6^]^3+^ (Table S2), albeit exhibiting somewhat slower exchange rates. Nevertheless, the low paramagnetic shift of the CEST peaks limits the potential use of this complex in future paraCEST MRI studies.

**Figure 8:**
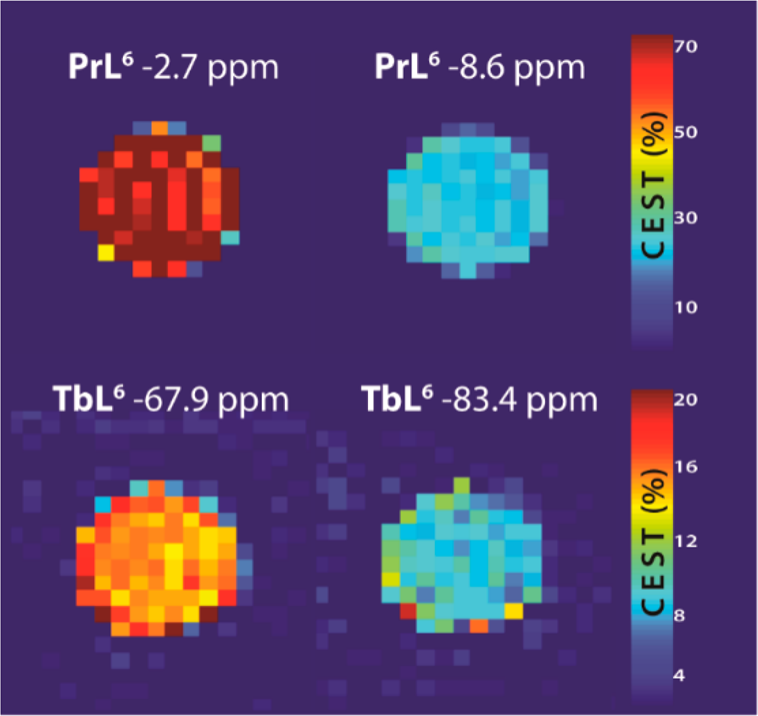
CEST MRI on tube phantoms with [PrL^6^]^3+^ and [TbL^6^]^3+^ (10 mM, 25 mMPBS, pH 7.0, RT, saturation time 5 s, saturation power 10 μT).

We also performed MRI experiments on the tube phantoms at 7 T magnetic field. We prepared the solutions of [TbL^6^]^3+^ and [PrL^6^]^3+^ in the same concentrations as those used in NMR experiments. We then obtained CEST MRI images using different saturation times and powers and compared the results to those obtained by means of NMR. Both complexes still showed strong CEST signals at room temperature (RT; Figure 8). However, because of the lower temperature in the MRI scanner (∼21–22 °C) and hence the slower exchange rates of amide protons during these experiments, the resulting CEST effect was lower (Table S2). Expectedly, the CEST MRI signals at a slightly shorter saturation time (3 s instead of 5 s) showed similar values when the saturation power was kept the same (10 μT), whereas a weaker saturation power (5 μT instead of 10 μT) resulted in much weaker CEST effects (Figure S13). Overall, the results obtained with [TbL^6^]^3+^ showed that it remains good candidate for further CEST MRI studies and is an excellent basis for the development of other potent paraCEST probes.

## Conclusions

The detailed structural investigation reported here evidences that the [LnL^6^]^3+^ complexes adopt different structures depending on the size of the lanthanide ion. The X-ray structure of the La^3+^ complex contains 12-coordinated metal ions, with the four pendant arms coordinating from the same side of the macrocyclic unit and a nitrate anion coordinating from the opposite side. However, NMR studies demonstrate that this unusual structure evolves to a 10-coordinated structure with an effective *D*_2_ symmetry in solution. The large Ln^3+^ ions adopt this structure in solution until Tb^3+^, while for the small ions, 9-coordinated structures are observed. The 10-coordinated structure of [TbL^6^]^3+^ was established by analyzing the paramagnetic shifts observed in the ^1^H NMR spectrum. The ^1^H and ^89^Y NMR spectra indicate that the major species present in solution is 9-coordinated, with a small proportion of the symmetrical *D*_2_ structure also being present (∼14%).

The [TbL^6^]^3+^ complex contains two pools of amide protons with relatively large chemical shifts showing strong temperature dependence (ranging from ∼69 to 85 ppm depending on the temperature). These amide protons are characterized by a rather slow exchange rate with bulk water (1.5–2.5 kHz). Furthermore, each of the CEST signals originates by four magnetically equivalent protons, which results in strong CEST responses even at low saturation powers. Thus, [TbL^6^]^3+^ can be regarded as a very attractive platform to develop CEST MRI agents.

## Experimental Section

### General Methods

^1^H NMR spectra were obtained at 25 °C using a Bruker ARX400 spectrometer and solutions of the complexes in D_2_O. Chemical shifts were referenced with respect to the residual HDO proton signal (δ = 4.79 ppm).^50^ Elemental analyses were obtained with a Carlo-Erba EA 1108 microanalyzer. Fourier transform infrared spectra were recorded using the attenuated total reflection method (ATR-FTIR) with a Bruker VECTOR 22 spectrometer (KBr disks). Mass spectra were obtained with a microTOF (focus) mass spectrometer (Bruker Daltonics, Bremen, Germany) equipped with an ApolloII (ESI) source for electrospray ionization.

### Preparation of the Complexes

All complexes were prepared by the reaction of a solution of Ln(NO_3_)_3_·*x*H_2_O (0.040 mmol) and L^6^·H_2_O (0.023 g, 0.040 mmol) in methanol (15 mL), following the same procedure as that described for the europium and yttrium analogues.^22^ Slow concentration of the solutions of the complexes in methanol yielded polycrystalline solids with the formula [LnL^6^](NO_3_)_3_·*x*H_2_O (*x* = 2–4) that were isolated by filtration and dried.

#### [LaL^6^](NO_3_)_3_·3H_2_O

L^6^·H_2_O (0.023 g, 0.040 mmol) and La(NO_3_)_3_·5H_2_O (0.017 g, 0.040 mmol) were reacted. Yield: 0.029 g, 79%. Elem anal. Calcd for C_26_H_44_N_13_O_16_La: C, 33.5; H, 4.8; N, 19.5. Found: C, 33.7; H, 5.0; N, 19.4. ESI-MS. Found (calcd): *m*/*z* 754.2 (754.2) for [La(L^6^-H)(NO_3_)]^+^, 691.2 (691.2) for [La(L^6^-2H)]^+^. IR (cm^–1^): 1600 (s), 1454 (s) [ν(C–C)/ν(C–C); pyridine], 1648 (s) [carbonyl stretch], 3165 (m) [ν(NH_2_)], 1293 (s), 817 (m), 827 (m), 732 (m) [N–O, nitrate].

#### [LaL^6^(NO_3_)]_2_[La(NO_3_)_6_]·NO_3_·4CH_3_OH

The synthesis followed the same procedure as that described above but using a 1.5:1 La(NO_3_)_3_·5H_2_O/L^6^ molar ratio. L^6^·H_2_O (0.023 g, 0.040 mmol) and La(NO_3_)_3_·5H_2_O (0.025 g, 0.060 mmol). Yield: 0.053 g, 60%. Elem anal. Calcd for C_56_H_92_N_29_O_39_La_3_: C, 30.4; H, 4.2; N, 18.4. Found: C, 30.5; H, 4.7; N, 18.8. MS (ESI^+^). Found (calcd): *m*/*z* 754.2 (754.2) for [La(L^6^-H)(NO_3_)]^+^, 691.2 (691.2) for [La(L^6^-2H)]^+^. IR (cm^–1^): 1594 (s), 1458 (s) [ν(C–C)/ν(C–C); pyridine], 1656 (s) [carbonyl stretch], 3280 (m) [ν(NH_2_)], 1296 (s), 818 (m), 731 (m) [N–O, nitrate]. Crystals suitable for X-ray diffraction were obtained with the formula [LaL^6^(NO_3_)]_2_[La(NO_3_)_6_]·NO_3_·4CH_3_OH.

#### [CeL^6^](NO_3_)_3_·3H_2_O

L^6^·H_2_O (0.023 g, 0.040 mmol) and Ce(NO_3_)_3_·6H_2_O (0.017 g, 0.040 mmol) were reacted. Yield: 0.024 g, 65%. Elem anal. Calcd for C_26_H_44_N_13_O_16_Ce: C, 33.4; H, 4.7; N, 19.5. Found: C, 33.6; H, 5.1; N, 19.4. MS (ESI^+^). Found (calcd): *m*/*z* 755.2 (755.2) for [Ce(L^6^-H)(NO_3_)]^+^, 692.2 (692.2) for [Ce(L^6^-2H)]^+^. IR (cm^–1^): 1593 (s), 1458 (s) [ν(C–C)/ν(C–C); pyridine], 1655 (s) [carbonyl stretch], 3283 (m) [ν(NH_2_)], 1240 (s), 1225 (m), 835 (m) [N–O, nitrate].

#### [PrL^6^](NO_3_)_3_·4H_2_O

L^6^·H_2_O (0.023 g, 0.040 mmol) and Pr(NO_3_)_3_·5H_2_O (0.017 g, 0.040 mmol) were reacted. Yield: 0.030 g, 80%. Elem anal. Calcd for C_26_H_46_N_13_O_17_Pr: C, 32.7; H, 4.8; N, 19.1. Found: C, 32.7; H, 5.0; N, 19.0. MS (ESI^+^). Found (calcd): *m*/*z* 756.2 (756.2) for [Pr(L^6^-H)(NO_3_)]^+^, 693.2 (693.2) for [Pr(L^6^-2H)]^+^. IR (cm^–1^): 1601 (s), 1460 (s) [ν(C–C)/ν(C–C); pyridine], 1654 (s) [carbonyl stretch], 3163 (m) [ν(NH_2_)], 1313 (s), 827 (m) [N–O, nitrate].

#### [NdL^6^](NO_3_)_3_·3H_2_O

L^6^·H_2_O (0.023 g, 0.040 mmol) and Nd(NO_3_)_3_·6H_2_O (0.017 g, 0.040 mmol) were reacted. Yield: 0.028 g, 75%. Elem anal. Calcd for C_26_H_44_N_13_O_16_Nd: C, 33.3; H, 4.7; N, 19.4. Found: C, 33.7; H, 5.0; N, 19.3. MS (ESI^+^). Found (calcd): *m*/*z* 757.2 (757.7) for [Nd(L^6^-H)(NO_3_)]^+^, 694.2 (694.2) for [Nd(L^6^-2H)]^+^. IR (cm^–1^): 1593 (s), 1458 (s) [ν(C–C)/ν(C–C); pyridine], 1655 (s) [carbonyl stretch], 3284 (m), 3225 (m) [ν(NH_2_)], 1299 (s), 838 (m), 734 (m) [N–O, nitrate].

#### [SmL^6^](NO_3_)_3_·3H_2_O

L^6^·H_2_O (0.023 g, 0.040 mmol) and Sm(NO_3_)_3_·6H_2_O (0.018 g, 0.040 mmol) were reacted. Yield: 0.032 g, 86%. Elem anal. Calcd for C_26_H_44_N_13_O_16_Sm: C, 33.0; H, 4.7; N, 19.3. Found: C, 32.9; H, 5.0; N, 19.4. MS (ESI^+^). Found (calcd): *m*/*z* 767.2 (766.2) for [Sm(L^6^-H)(NO_3_)]^+^, 704.2 (704.2) for [Sm(L^6^-2H)]^+^. IR (cm^–1^): 1594 (s), 1458 (s) [ν(C–C)/ν(C–C); pyridine], 1656 (s) [carbonyl stretch], 3158 (m) [ν(NH_2_)], 1295 (s), 842 (m), 736 (m) [N–O, nitrate]. Crystals suitable for X-ray diffraction with the formula [SmL^6^](NO_3_)_2.91_·Br_0.09_ were obtained from a solution of the complex in water after evaporation at RT.

#### [GdL^6^](NO_3_)_3_·4H_2_O

L^6^·H_2_O (0.023 g, 0.040 mmol) and Gd(NO_3_)_3_·6H_2_O (0.018 g, 0.040 mmol) were reacted. Yield: 0.028 g, 72%. Elem anal. Calcd for C_26_H_46_N_13_O_17_Gd: C, 32.2; H, 4.8; N, 18.8. Found: C, 32.7; H, 5.0; N, 18.4. MS (ESI^+^). Found (calcd): *m*/*z* 773.2 (773.2) for [Gd(L^6^-H)(NO_3_)]^+^, 710.2 (710.2) for [Gd(L^6^-2H)]^+^. IR (cm^–1^): 1593 (s), 1455 (s) [ν(C–C)/ν(C–C); pyridine], 1664 (s) [carbonyl stretch], 3277 (m), 3215 (m) [ν(NH_2_)], 1294 (s), 814 (m), 741 (m) [N–O, nitrate].

#### [TbL^6^](NO_3_)_3_·3H_2_O

L^6^·H_2_O (0.023 g, 0.040 mmol) and Tb(NO_3_)_3_·6H_2_O (0.018 g, 0.040 mmol) were reacted. Yield: 0.027 g, 70%. Elem anal. Calcd for C_26_H_44_N_13_O_16_Tb: C, 32.7; H, 4.6; N, 19.1. Found: C, 32.7; H, 4.8; N, 19.0. MS (ESI^+^). Found (calcd): *m*/*z* 774.2 (774.2) for [Tb(L^6^-H)(NO_3_)]^+^, 711.2 (711.2) for [Tb(L^6^-2H)]^+^. IR (cm^–1^): 1590 (s), 1456 (s) [ν(C–C)/ν(C–C); pyridine], 1655 (s) [carbonyl stretch], 3160 (m) [ν(NH_2_)], 1315 (s), 828 (m) [N–O, nitrate].

#### [DyL^6^](NO_3_)_3_·2H_2_O

L^6^·H_2_O (0.023 g, 0.040 mmol) and Dy(NO_3_)_3_·5H_2_O (0.017 g, 0.040 mmol) were reacted. Yield: 0.026 g, 68%. Elem anal. Calcd for C_26_H_42_N_13_O_15_Dy: C, 33.2; H, 4.5; N, 19.4. Found: C, 33.0; H, 4.8; N, 19.4. MS (ESI^+^). Found (calcd): *m*/*z* 779.2 (779.2) for [Dy(L^6^-H)(NO_3_)]^+^, 716.2 (716.2) for [Dy(L^6^-2H)]^+^. IR (cm^–1^): 1589 (s), 1459 (s) [ν(C–C)/ν(C–C); pyridine], 1655 (s) [carbonyl stretch], 3176 (m) [ν(NH_2_)], 1281 (s), 813 (m), 744 (m) [N–O, nitrate].

#### [HoL^6^](NO_3_)_3_·3H_2_O

L^6^·H_2_O (0.023 g, 0.040 mmol) and Ho(NO_3_)_3_·5H_2_O (0.017 g, 0.040 mmol) were reacted. Yield: 0.023 g, 59%. Elem anal. Calcd for C_26_H_44_N_13_O_16_Ho: C, 32.5; H, 4.6; N, 19.0. Found: C, 32.7; H, 4.8; N, 18.8. MS (ESI^+^). Found (calcd): *m*/*z* 780.2 (780.2) for [Ho(L^6^-H)(NO_3_)]^+^, 717.2 (717.2) for [Ho(L^6^-2H)]^+^. IR (cm^–1^): 1591 (s), 1459 (s) [ν(C–C)/ν(C–C); pyridine], 1660 (s) [carbonyl stretch], 3168 (m) [ν(NH_2_)], 1324 (s), 827 (m), 779 (m) [N–O, nitrate].

#### [ErL^6^](NO_3_)_3_·3H_2_O

L^6^·H_2_O (0.023 g, 0.040 mmol) and Er(NO_3_)_3_·5H_2_O (0.017 g, 0.040 mmol) were reacted. Yield: 0.026 g, 68%. Elem anal. Calcd for C_26_H_44_N_13_O_16_Er: C, 32.5; H, 4.6; N, 19.0. Found: C, 32.3; H, 4.9; N, 19.1. MS (ESI^+^). Found (calcd): *m*/*z* 782.2 (781.4) for [Er(L^6^-H)(NO_3_)]^+^, 718.2 (718.2) for [Er(L^6^-2H)]^+^. IR (cm^–1^): 1591 (s), 1459 (s) [ν(C–C)/ν(C–C); pyridine], 1655 (s) [carbonyl stretch], 3171 (m) [ν(NH_2_)], 1294 (s), 811 (m), 746 (m) [N–O, nitrate].

#### [TmL^6^](NO_3_)_3_·4H_2_O

L^6^·H_2_O (0.023 g, 0.040 mmol) and Tm(NO_3_)_3_·5H_2_O (0.018 g, 0.040 mmol) were reacted. Yield: 0.030 g, 78%. Elem anal. Calcd for C_26_H_46_N_13_O_17_Tm: C, 31.8; H, 4.7; N, 18.6. Found: C, 31.9; H, 4.6; N, 18.4%. MS (ESI^+^). Found (calcd): *m*/*z* 784.2 (784.2) for [Tm(L^6^-H)(NO_3_)]^+^, 721.2 (721.2) for [Tm(L^6^-2H)]^+^. IR (cm^–1^): 1606 (s), 1460 (s) [ν(C–C)/ν(C–C); pyridine], 1655 (s) [carbonyl stretch], 3177 (m) [ν(NH_2_)], 1288 (s), 812 (m), 748 (m) [N–O, nitrate].

#### [YbL^6^](NO_3_)_3_·3H_2_O

L^6^·H_2_O (0.023 g, 0.040 mmol) and Yb(NO_3_)_3_·5H_2_O (0.018 g, 0.040 mmol) were reacted. Yield: 0.027 g, 69%. Elem anal. Calcd for C_26_H_44_N_13_O_16_Yb: C, 32.3; H, 4.8; N, 18.8. Found: C, 32.3; H, 5.0; N, 18.5. MS (ESI^+^). Found (calcd): *m*/*z* 789.2 (789.2) for [Yb(L^6^-H)(NO_3_)]^+^, 726.2 (726.2) for [Yb(L^6^-2H)]^+^. IR (cm^–1^): 1592 (s), 1460 (s) [ν(C–C)/ν(C–C); pyridine], 1664 (s) [carbonyl stretch], 3175 (m) [ν(NH_2_)], 1325 (s), 826 (m) [N–O, nitrate]. Crystals of [YbL^6^](NO_3_)_2.7_·Br_0.3_·3H_2_O suitable for X-ray diffraction measurements were obtained from a solution of the complex in water, which was left to evaporate slowly at RT.

#### [LuL^6^](NO_3_)_3_·4H_2_O

L^6^·H_2_O (0.023 g, 0.040 mmol) and Lu(NO_3_)_3_·H_2_O (0.015 g, 0.040 mmol) were reacted. Yield: 0.032 g, 80%. Elem anal. Calcd for C_26_H_46_N_13_O_17_Lu: C, 31.6; H, 4.8; N, 18.4. Found: C, 31.7; H, 5.1; N, 18.2. MS (ESI^+^). Found (calcd): *m*/*z* 790.2 (790.2) for [Lu(L^6^-H)(NO_3_)]^+^, 727.2 (727.2) for [Lu(L^6^-2H)]^+^. IR (cm^–1^): 1599 (s), 1460 (s) [ν(C–C)/ν(C–C); pyridine], 1654 (s) [carbonyl stretch], 3177 (m) [ν(NH_2_)], 1294 (s), 812 (m), 751 (m) [N–O, nitrate].

### Crystal Structure Determinations

X-ray diffraction data of [LaL^6^(NO_3_)]_2_[La(NO_3_)_6_]·NO_3_·4CH_3_OH and [SmL^6^](NO_3_)_2.91_·Br_0.09_ were recorded at 273(2) K using a Bruker Smart-CCD-1000 diffractometer and graphite-monochromated Mo Kα radiation (λ = 0.71073 Å). Corrections for Lorentz and polarization effects were applied to all data. For [YbL^6^](NO_3_)_2.7_·Br_0.3_·3H_2_O, X-ray diffraction data were obtained at 100(2) K with Mo Kα radiation and a Bruker D8 Venture Photon 100 CMOS detector. Collection of frames of data, reflection indexing, and lattice parameter determination were achieved with the *APEX3* software. Integration of the intensity of the reflections was carried out with *SAINT*.^51^ The software *SADABS*(52) was used in all cases for scaling and empirical absorption correction. All structures were refined by full-matrix least squares based on *F*^2^ with the *SHELXT* program.^53^ Non-H atoms were refined with anisotropic displacement parameters. H atoms were included in calculated positions and refined with isotropic displacement parameters. For [SmL^6^](NO_3_)_2.91_·Br_0.09_, a solvent masking routine was applied to correct the reflection data for the diffuse scattering associated with disordered water molecules present in the crystal. Molecular graphics were generated using *OLEX2*.^54^ Crystal data and details on data refinement are provided in Table 5.

**Table 5:** Crystallographic and Structure Refinement Data for [LaL^6^(NO_3_)]_2_[La(NO_3_)_6_]·NO_3_·4CH_3_OH, [SmL^6^](NO_3_)_2.91_·Br_0.09_, and [YbL^6^](NO_3_)_2.7_·Br_0.3_·3H_2_O

formula	C_28_H_46_N_14.5_O_19.5_ La_1.5_	C_26_H_37_N_12.91_O_12.74_Br_0.09_Sm	C_26_H_44_N_12.71_O_15.13_Br_0.29_Yb
MW	1106.16	891.56	972.94
cryst syst	monoclinic	monoclinic	triclinic
space group	*C*2/*c*	*C*2	*P*1̅
*a*/Å	27.765(3)	24.934(3)	10.2831(7)
*b*/Å	21.204(2)	12.0748(13)	10.4226(7)
*c*/Å	17.5621(19)	24.057(3)	7.7173(12)
α/deg			85.062(2)
β/deg	125.119(2)	91.131(2)	87.922(2)
γ/deg			75.771(2)
*V*/Å^3^	8457.1(16)	7241.4(13)	1833.6(2)
*Z*	8	8	2
*D*_calc_/g cm^–3^	1.738	1.636	1.762
μ/mm^–1^	1.596	1.795	2.951
Flack parameter		0.381(17)	
*R*_int_	0.0657	0.0509	0.0324
R1^a^	0.0564	0.0477	0.0204
wR2 (all data)^b^	0.1632	0.1158	0.0432

a R1 = ∑||*F*_o_| – |*F*_c_||/∑|*F*_o_|.

b wR2 = {∑[*w*(||*F*_o_|^2^ – |*F*_c_|^2^|)^2^]/∑[*w*(*F*_o_^4^)]}^1/2^.

### CEST Studies

NMR experiments of [PrL^6^]^3+^ and [TbL^6^]^3+^ (10 mM, pH 7.0, PBS 25 mM) were recorded using a 300 MHz Bruker Avance III NMR instrument (Bruker, Ettlingen, Germany). The recordings were done using a saturation time of 8 s at different temperatures (25 and 37 °C for [PrL^6^]^3+^ and 10, 15, 20, 25, 30, 35, 37, and 40 °C for [TbL^6^]^3+^). Variable saturation powers (*B*_1_ = 2.5, 5, 10, 15, 20, 25, and 30 μT) were used for each temperature, employing a constant saturation time. The longitudinal (*T*_1_) and transverse (*T*_2_) relaxation times were determined with the standard inversion–recovery and Carr–Purcell–Meiboom–Gill pulse sequences, respectively.^55,56^ The exchange rates *k*_ex_ were assessed with the BM equations.^47^

MRI measurements were recorded from tube phantoms with a 300 MHz Bruker 70/30 USR magnet and a Bruker volume coil (RF RES 300 1H 075/040 QSN TR). Images were processed with *Paravision 5.1* software provided by Bruker. MRI phantoms consisted of 2 × 400 μL vials placed inside the holder, inserted in the 60 mL syringe filled with water. The vials contained 10 mM [TbL^6^]^3+^ and [PrL^6^]^3+^ (pH 7.0, 25 mM PBS), respectively.

CEST MRI images were acquired at RT using rapid acquisition with relaxation enhancement (RARE) pulse sequence with the following imaging parameters: repetition time/echo time = 16280/3.26 ms, field of view = 32 × 32 mm, matrix size = 64 × 64, slice thickness = 2 mm, rare factor = 64, number of excitation = 1, acquisition time = 35 min 49 s. MT parameters were as follows: saturation pulse duration 3 and 5 s, saturation power 5 and 10 μT, 131 irradiation offsets in the range −100 to +100 ppm.

Image analysis was performed in *MATLAB* (MathWorks, USA). Initially, CEST MRI images were linearly interpolated and shifted to the center frequency in order to remove *B*_0_ inhomogeneity artifacts. Thereafter, pairs of Z-spectrum images were extracted for the irradiation offsets of the CEST peaks (+Δω) for the complexes (−3 and −8.5 ppm for [PrL^6^]^3+^ and −68.5 and −85.0 ppm for [TbL^6^]^3+^), and their corresponding opposites (−Δω) with respect to bulk water.

Quantification of the CEST effect was achieved using an inverse asymmetry analysis of the normalized Z-magnetization, using the inverse difference of the magnetization transfer (MTR_ind_ in eq 5). The latter is calculated from the unsaturated water magnetization (*M*_0_) and magnetizations of the on-resonance at the frequency +Δω (*M*_*z*+_) and the off-resonance at −Δω (*M*_*z*–_) with respect to the bulk water signal.^57^
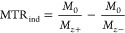
5

### Computational Details

Full geometry optimization of the [GdL^6^]^3+^ complex and the subsequent frequency analysis were carried out within the framework of DFT (TPSSh^58^ exchange correlation functional) employing the *Gaussian 09* package (revision D.01).^59^ The inner electrons of Gd (46 + 4f^7^) were described with a large-core quasi-relativistic effective core potential (RECP), while a [5s4p3d]-GTO basis set was used for the outermost 11 electrons.^60^ All other atoms were described using the 6-31G(d,p) basis set. Hyperfine coupling constants (*A*/ℏ) were calculated using a small-core relativistic effective core potential, which places 28 electrons of Gd in the core. The valence space of Gd was described with the ECP28MWB_GUESS basis set,^61^ while the EPR-III^62^ basis set was used for the ligand atoms. The effects of bulk water were incorporated in all calculations using the integral equation formalism of the polarized continuum model.^63^ The Y^3+^ complexes were optimized using a similar approach, with the TPSSh functional, the ECP28MWB quasi-relativistic ECP, and its associated basis set for Y and the standard 6-311G(d,p) basis set for all other atoms.^64^ The ^89^Y NMR shielding tensors were calculated with the GIAO^65^ method and the TPSSh functional,^58^ using the *ORCA* program package (version 4.2.0)^66^ and the DKH2^67^ relativistic method. In these calculations, we used the old-DKH-TZVPP basis set, as implemented in previous versions of *ORCA* (see the Supporting Information), which is based on the TZVPPAll basis set of Aldrich^68^ and was recontracted for DKH2 calculations. The resolution of identity approximation for both Coulomb- and exchange-type integrals (RIJK) was used for both self-consistent field and calculation of the NMR chemical shielding constants.^69,70^ Auxiliary basis sets were generated with the Autoaux procedure implemented in *ORCA*.^71^ Bulk solvents were considered using the SMD solvation model.^72^

## Supporting Information Available

Coordination polyhedra, NMR spectra, Cartesian coordinates obtained with DFT, calculated pseudocontact shifts, Eyring plots, and additional CEST data (PDF)

## Accession Codes

CCDC 2044164–2044166 contain the supplementary crystallographic data for this paper. These data can be obtained free of charge via www.ccdc.cam.ac.uk/data_request/cif, or by emailing data_request@ccdc.cam.ac.uk, or by contacting The Cambridge Crystallographic Data Centre, 12 Union Road, Cambridge CB2 1EZ, UK; fax: +44 1223 336033.
