# Supplementary material for: Lanthanide(III) Complexes Based on an 18-Membered Macrocycle Containing Acetamide Pendants. Structural Characterization and paraCEST Properties
Source: Inorg Chem. 2021 Jan 20;60(3):1902–14. doi: 10.1021/acs.inorgchem.0c03385 (PMC8929667; doi:10.1021/acs.inorgchem.0c03385)
Supplement: Supplementary file 1 — ic0c03385_si_001.pdf [file ic0c03385_si_001.pdf]

Supporting Information for:

Lanthanide(III) Complexes Based on a 18-Membered Macrocycle Containing Acetamide Pendants. Structural Characterization and paraCEST Properties

Goretti Castro,† Gaoji Wang,§ Tanja Gambino,§ David Esteban-Gómez,‡ Laura Valencia,† Goran Angelovski,§ Carlos Platas-Iglesias,*,‡ and Paulo Pérez-Lourido *,†

† Departamento de Química Inorgánica, Facultad de Ciencias, Universidade de Vigo, As Lagoas, Marcosende, 36310 Pontevedra, Spain

§ MR Neuroimaging Agents, Max Planck Institute for Biological Cybernetics, 72076 Tübingen, Germany

‡ Centro de Investigacións Científicas Avanzadas (CICA) and Departamento de Química, Universidade da Coruña, Campus da Zapateira-Rúa da Fraga 10, 15008 A Coruña, Spain

Email: carlos.platas.iglesias@udc.es (C. P.-I.)
Email: paulo@uvigo.es (P. P.L.)

Summary

Figure S1. View of the structure of the $[La(NO_3)_6]^{3-}$ anion present in crystals of $[LaL^6(NO_3)]_2[La(NO_3)_6]\cdot NO_3\cdot 4CH_3OH$. The ORTEP plot is at the 30% probability level. Bond distances of the metal coordination environment (Å): La(2)—O(8N), 2.632(7); La(2)—O(11N), 2.640(6); La(2)—O(12N), 2.643(6); La(2)—O(4N), 2.643(7); La(2)—O(10N), 2.672(7); La(2)—O(6N), 2.721(6). 4

Figure S2. View of the coordination polyhedron in $[LaL^6]^{3+}$ with the shape of a icosahedron. ..5

Figure S3. View of the coordination polyhedron in $[SmL^6]^{3+}$ with the shape of a sphenocorona. 6

Figure S4. View of the coordination polyhedron in $[YbL^6]^{3+}$ with the shape of a tricapped trigonal prism. 7

Figure S5. ^1H-^1H COSY spectrum of $[CeL^6]^{3+}$ recorded in D_2O solution (400 MHz, pH = 7.0, 25 ºC). The major signals correspond to the species with D_2 symmetry. 8

Figure S6. ^1H-^1H COSY spectrum of $[PrL^6]^{3+}$ recorded in D_2O solution (400 MHz, pH = 7.0, 25 ºC). 9

Figure S7. ^1H-^1H COSY spectrum of $[NdL^6]^{3+}$ recorded in D_2O solution (400 MHz, pH = 7.0, 25 ºC). 10

Figure S8. ^1H-^1H COSY spectrum of $[SmL^6]^{3+}$ recorded in D_2O solution (400 MHz, pH = 7.0, 25 ºC). 11

Figure S9. Structure of the $[GdL^6]^{3+}$ complex obtained with DFT calculations. 12

Figure S10. ^1H-^1H COSY spectrum of $[YbL^6]^{3+}$ recorded in D_2O solution (400 MHz, pH = 7.0, 25 ºC). 13

Figure S11. Structure of the $[Y(H_2O)_8]^{3+}\cdot 16H_2O$ system optimized with DFT calculations. 14

Figure S12. Z-spectra recorded at 7 T of a solution containing $[PrL^6]^{3+}$ (10 mM in 25 mM PBS, pH 7.0). Saturation time 8 s, saturation power B_1 = 5 µT at 25 and 37 °C. 15

Figure S13. CEST MRI on tube phantoms with $[PrL^6]^{3+}$ and $[TbL^6]^{3+}$ (10 mM, 25 mM PBS, pH 7.0, RT): saturation time 5 s, saturation power 5 µT (left), saturation time 3 s, saturation power 10 µT (right). 15

Figure S14. Eyring plot for the exchange of amide protons in $[TbL^6]^{3+}$. The most shifted proton is represented as blue squares, and the signal with the smallest shift as orange circles. 16

Table S1. Comparison of the experimental and calculated ^1H NMR shifts obtained for the $[TbL^6]^{3+}$ complex. The calculated shifts were obtained as $\delta^{obs} = \delta^{dia} + \delta^{con} + \delta^{pscon}$, with pseudocontact shifts obtained from the fit to Eq (3). 17

Table S2. Exchange rate values and frequency shifts obtained from the BM multi-B1 fitting procedures for $[TbL^6]^{3+}$ and $[PrL^6]^{3+}$. 17

Table S3. CEST MRI on tube phantoms with **PrL⁶** and **TbL⁶** (10 mM, 25 mM PBS, pH 7.0, RT). Comparison of CEST effects under different MT parameters. 18

Table S4. Cartesian coordinates optimized for the $[Y(H_2O)_8]^{3+}\cdot 16H_2O$ system (0 imaginary frequencies). 18

Table S5. Cartesian coordinates optimized for the $[YL^6]^{3+}\cdot 16H_2O$ system (0 imaginary frequencies, CN 9). 20

Table S6. Cartesian coordinates optimized for the $[YL^6]^{3+}\cdot 16H_2O$ system (0 imaginary frequencies, CN 10). 21

Table S7. Old-DKH-TZVPP basis set used for ^{89}Y NMR shielding tensor calculations. 23

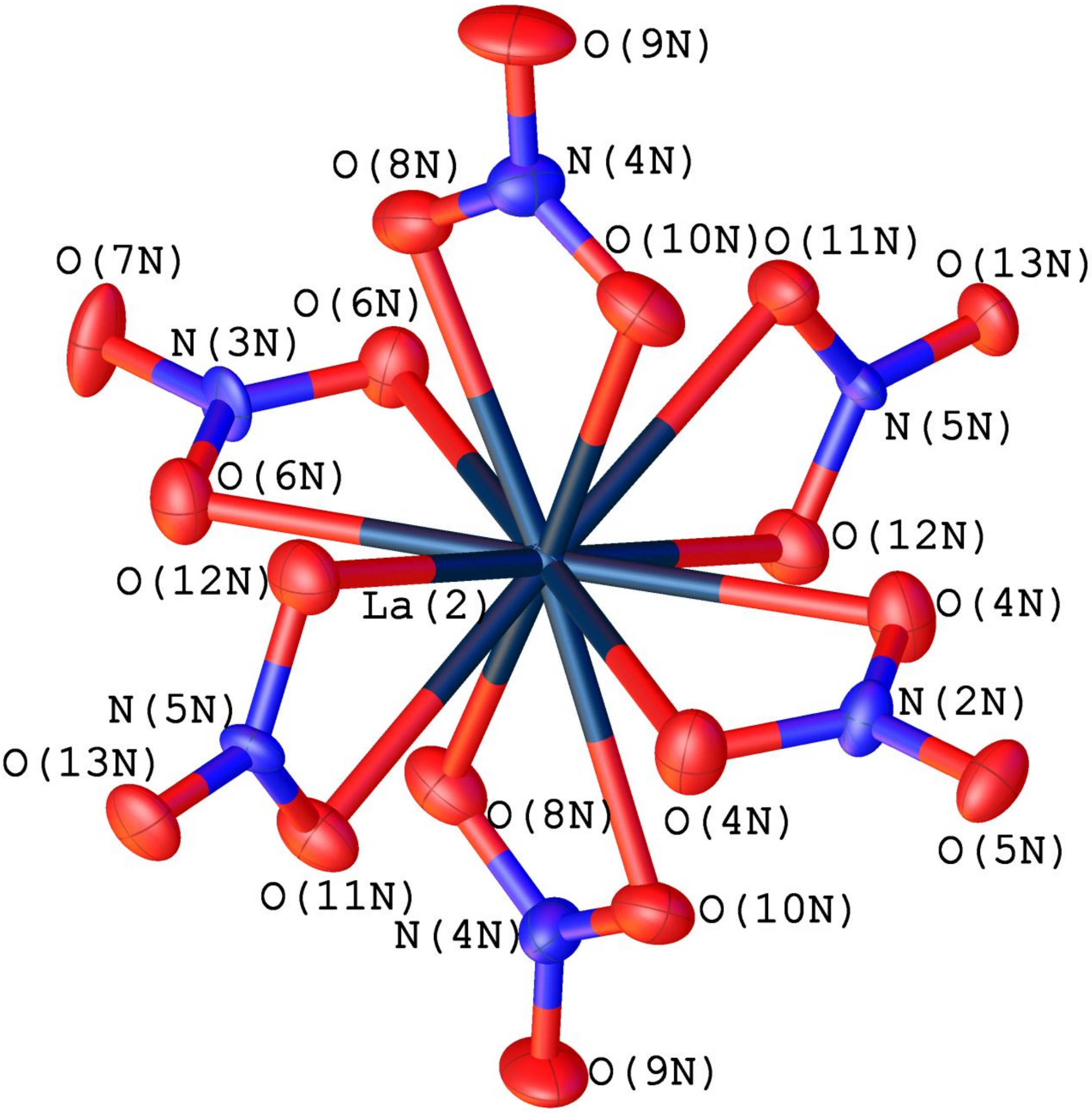

Figure S1. View of the structure of the $[La(NO_3)_6]^{3-}$ anion present in crystals of $[LaL^6(NO_3)]_2[La(NO_3)_6]\cdot NO_3\cdot 4CH_3OH$. The ORTEP plot is at the 30% probability level. Bond distances of the metal coordination environment (Å): La(2)—O(8N), 2.632(7); La(2)—O(11N), 2.640(6); La(2)—O(12N), 2.643(6); La(2)—O(4N), 2.643(7); La(2)—O(10N), 2.672(7); La(2)—O(6N), 2.721(6).

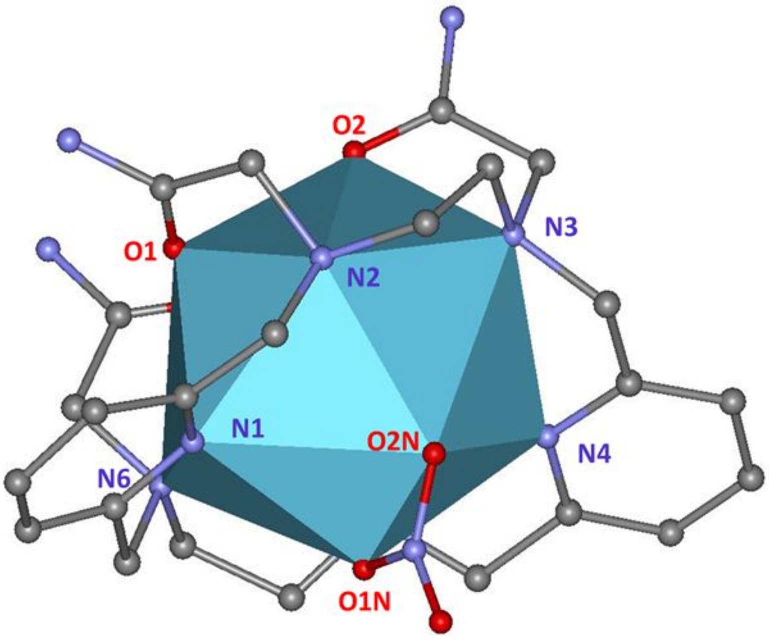

Figure S2. View of the coordination polyhedron in $[LaL^6]^{3+}$ with the shape of a icosahedron.

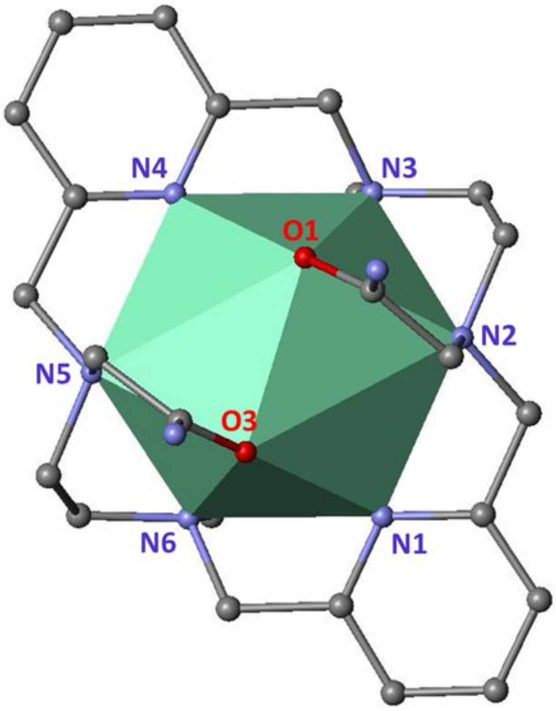

Figure S3. View of the coordination polyhedron in $[SmL^6]^{3+}$ with the shape of a sphenocorona.

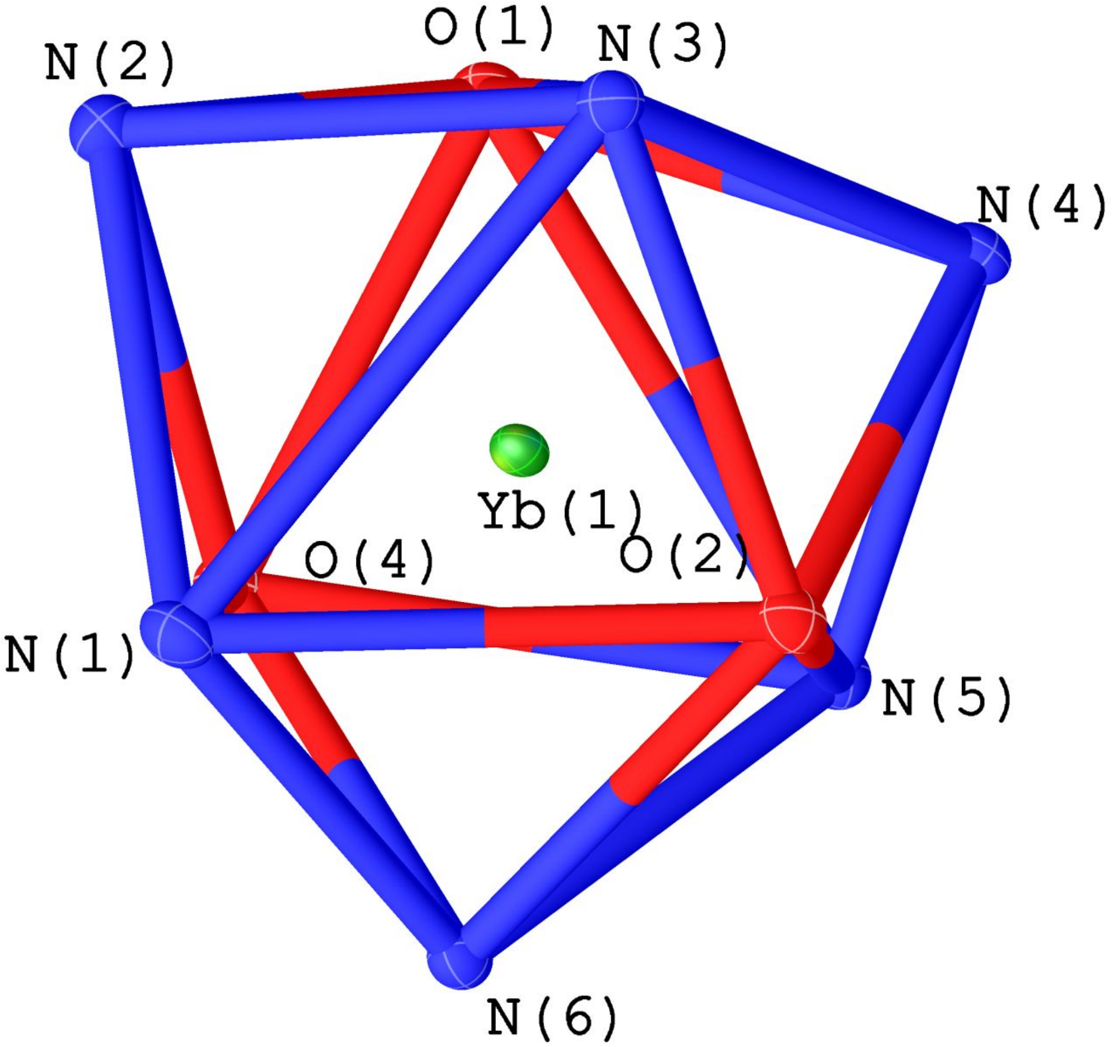

Figure S4. View of the coordination polyhedron in $[YbL^6]^{3+}$ with the shape of a tricapped trigonal prism.

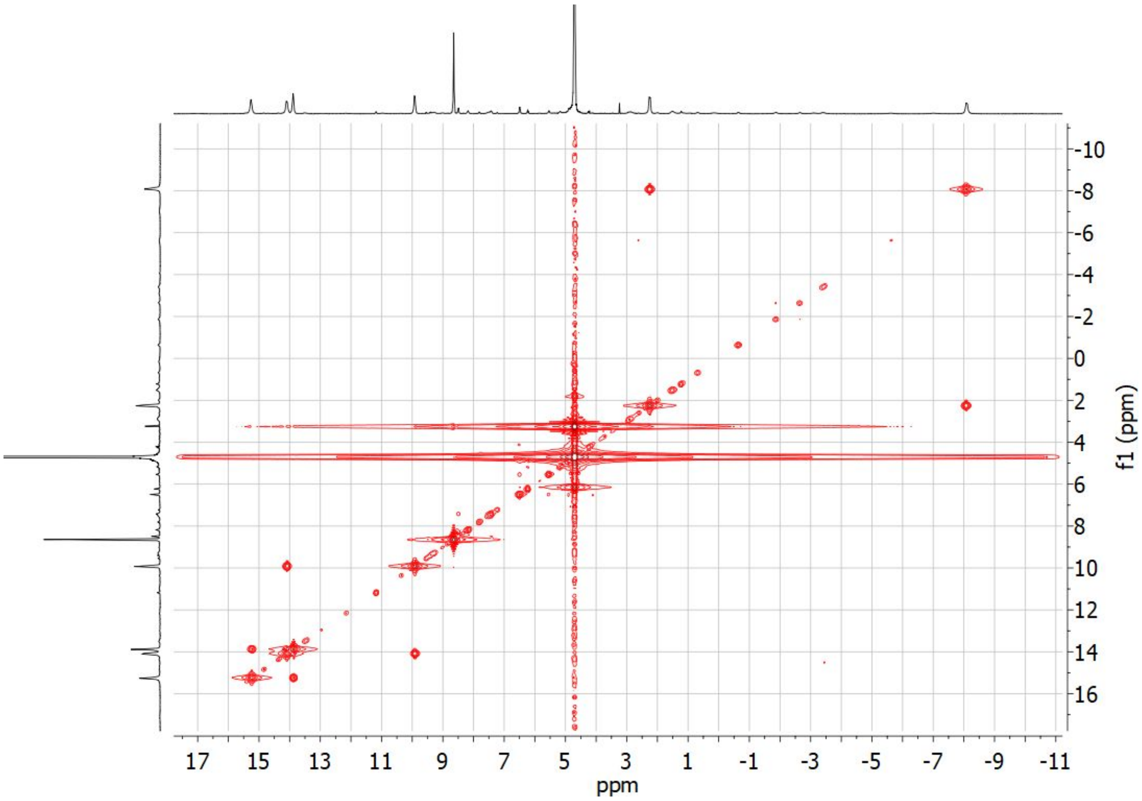

Figure S5. ^{1}H-^{1}H COSY spectrum of $[CeL^{6}]^{3+}$ recorded in D_2O solution (400 MHz, pH = 7.0, 25 ºC). The major signals correspond to the species with D_2 symmetry.

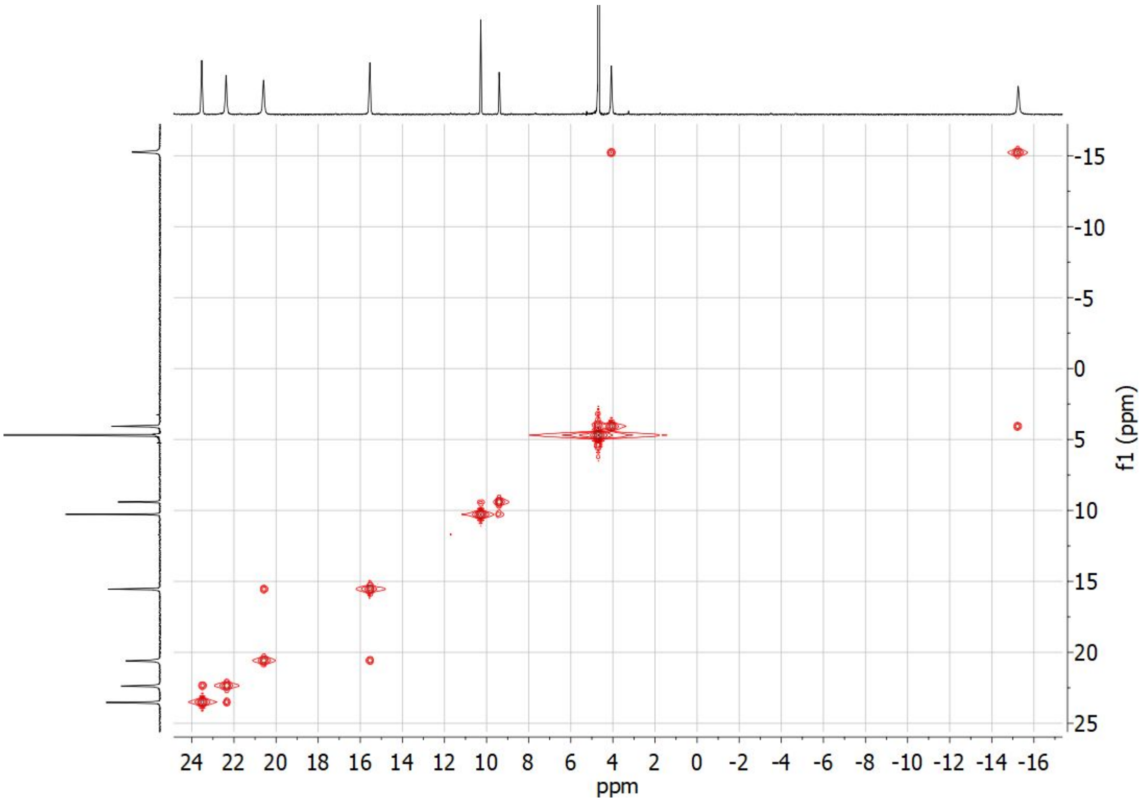

Figure S6. ^{1}H-^{1}H COSY spectrum of $[PrL^6]^{3+}$ recorded in D_2O solution (400 MHz, pH = 7.0, 25 ºC).

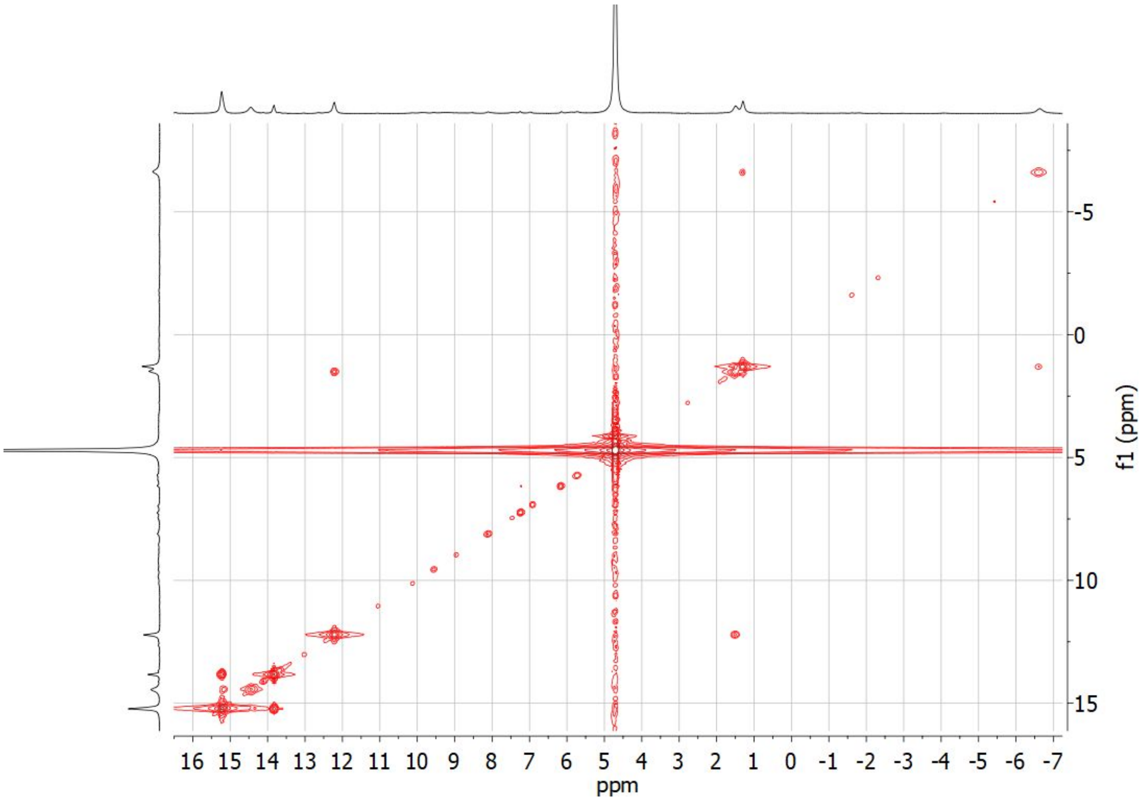

Figure S7. ^1H-^1H COSY spectrum of $[NdL^6]^{3+}$ recorded in D_2O solution (400 MHz, pH = 7.0, 25 ºC).

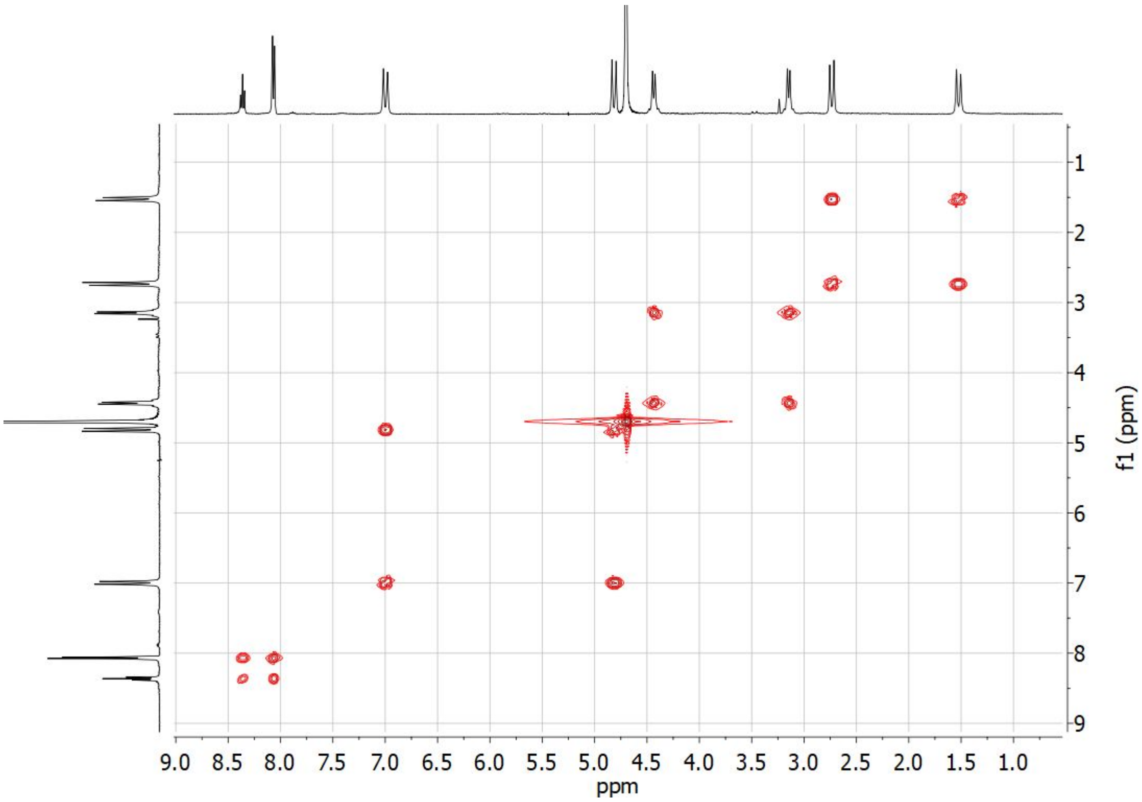

Figure S8. ^1H-^1H COSY spectrum of $[SmL^6]^{3+}$ recorded in D_2O solution (400 MHz, pH = 7.0, 25 ºC).

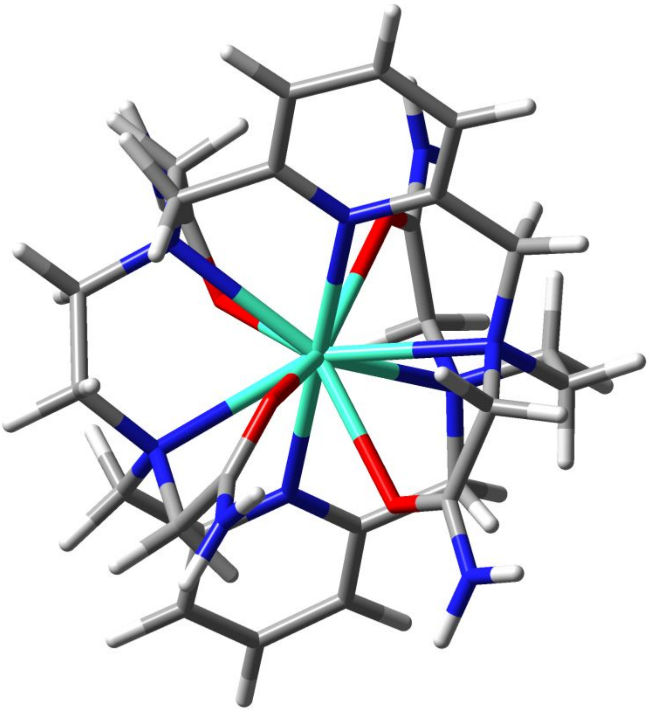

Figure S9. Structure of the $[GdL^6]^{3+}$ complex obtained with DFT calculations.

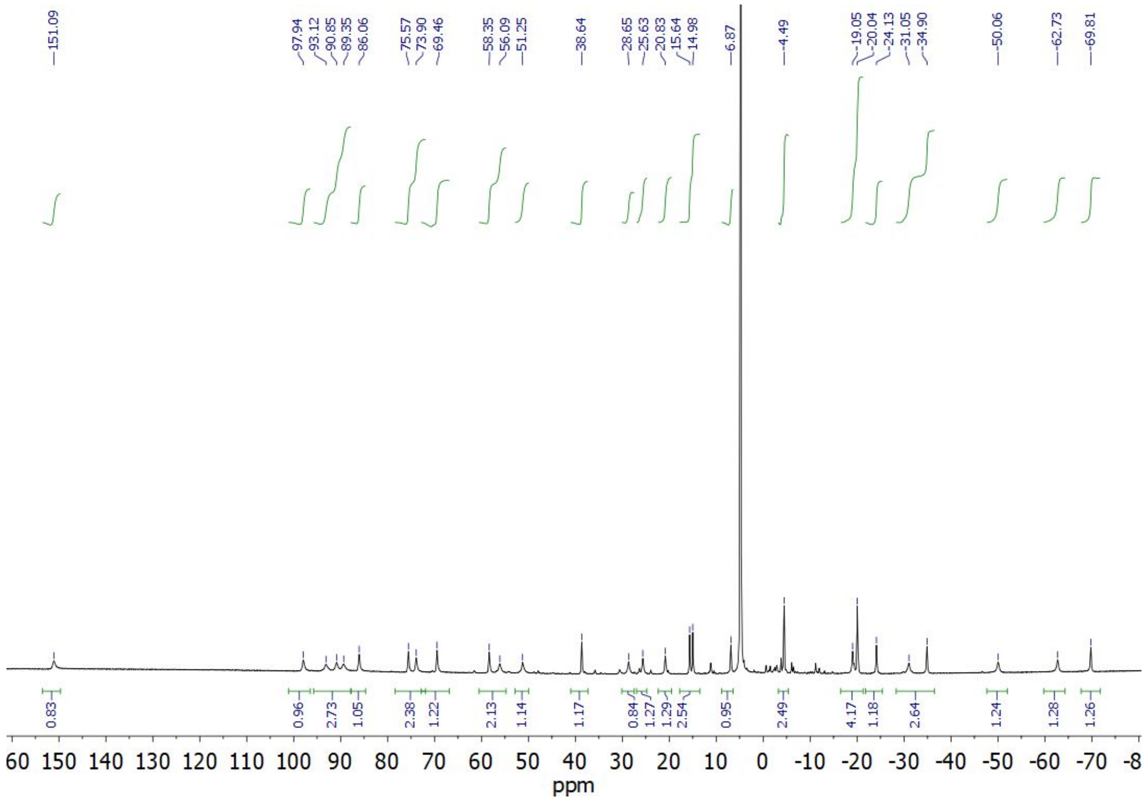

Figure S10. ^{1}H-^{1}H COSY spectrum of $[YbL^{6}]^{3+}$ recorded in D_2O solution (400 MHz, pH = 7.0, 25 ºC).

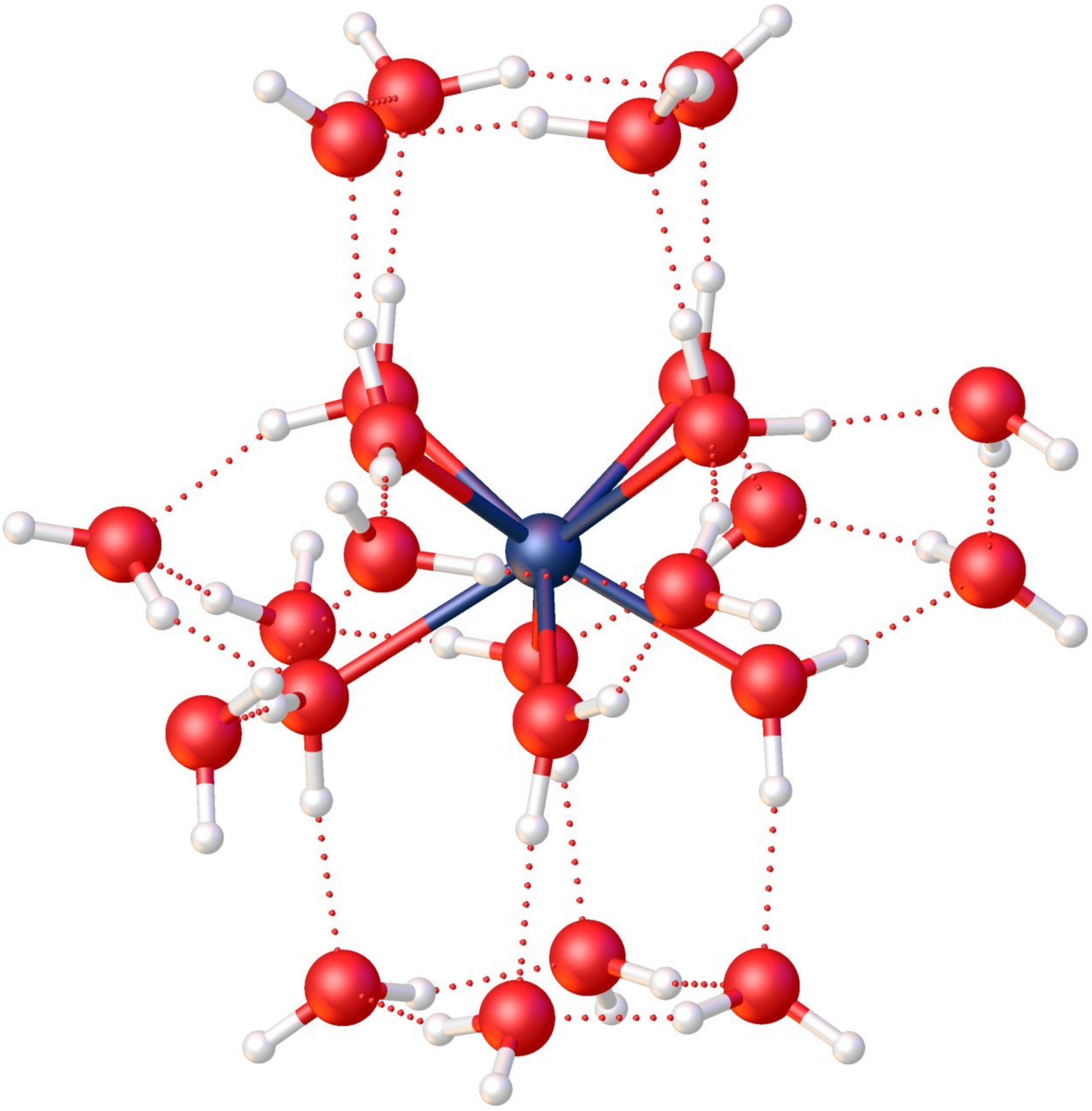

Figure S11. Structure of the $[Y(H_2O)_8]^{3+}\cdot 16H_2O$ system optimized with DFT calculations.

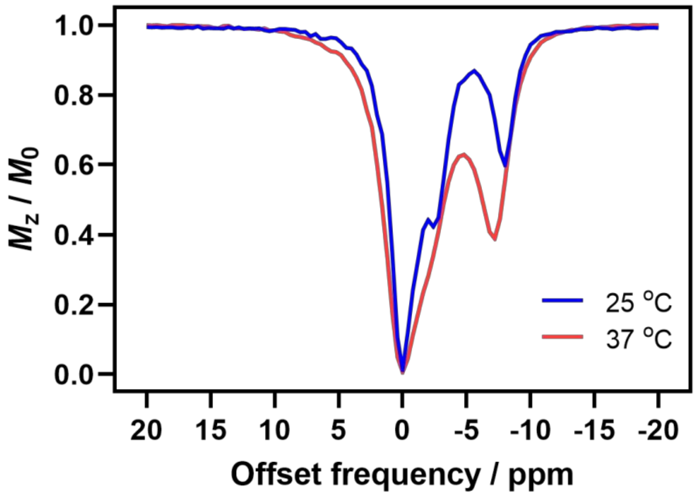

Figure S12. *Z*-spectra recorded at 7 T of a solution containing $[PrL^6]^{3+}$ (10 mM in 25 mM PBS, pH 7.0). Saturation time 8 s, saturation power B_1 = 5 µT at 25 and 37 °C.

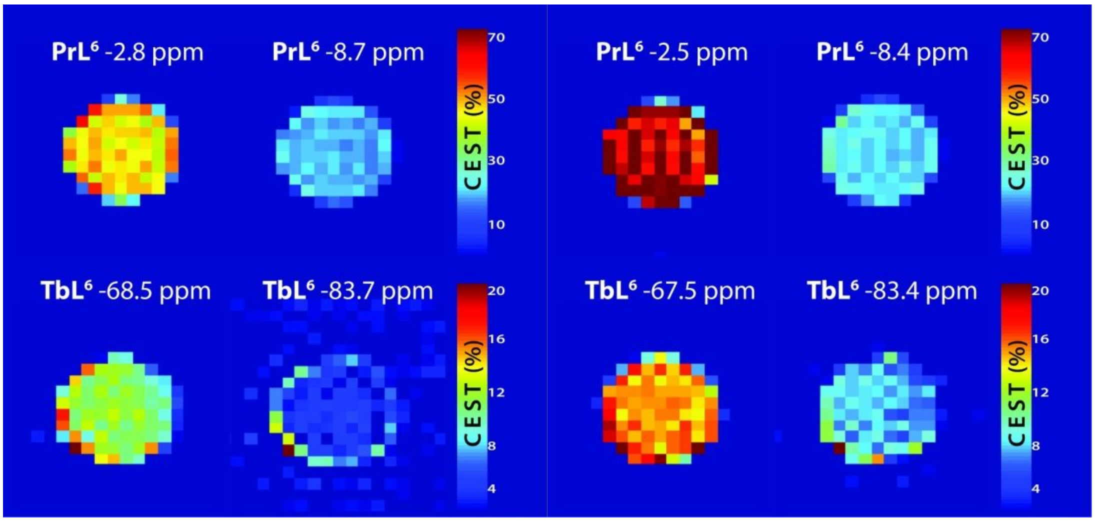

Figure S13. CEST MRI on tube phantoms with $[PrL^6]^{3+}$ and $[TbL^6]^{3+}$ (10 mM, 25 mM PBS, pH 7.0, RT): saturation time 5 s, saturation power 5 µT (left), saturation time 3 s, saturation power 10 µT (right).

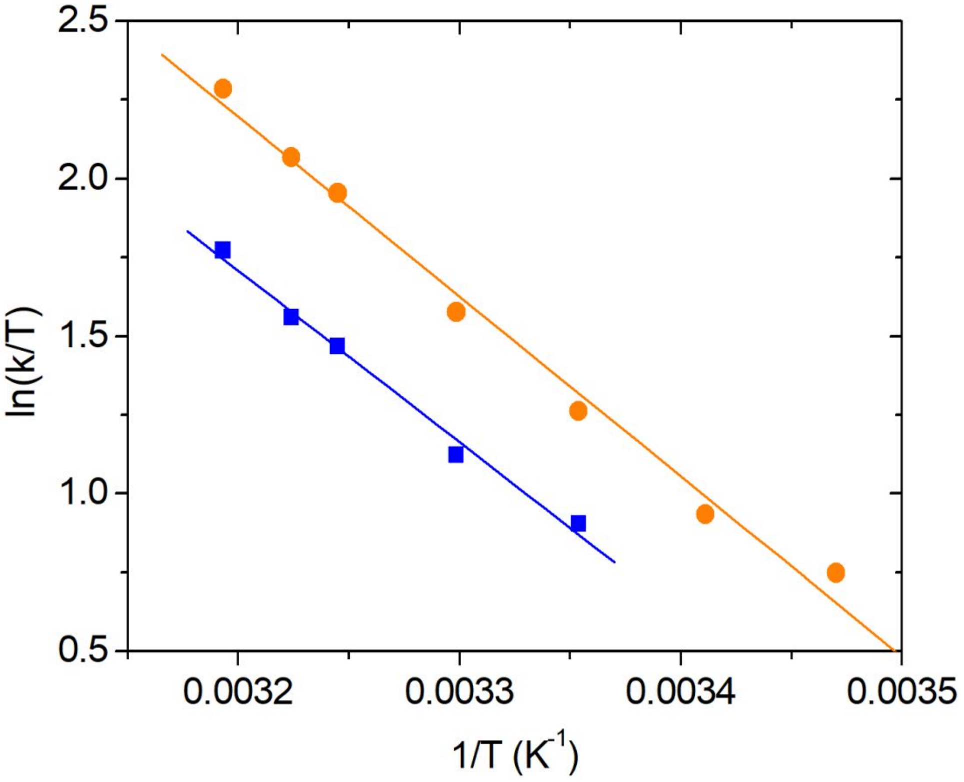

Figure S14. Eyring plot for the exchange of amide protons in $[TbL^6]^{3+}$. The most shifted proton is represented as blue squares, and the signal with the smallest shift as orange circles.

Table S1. Comparison of the experimental and calculated ^{1}H NMR shifts obtained for the $[TbL^6]^{3+}$ complex. The calculated shifts were obtained as $\delta^{obs} = \delta^{dia} + \delta^{con} + \delta^{pscon}$, with pseudocontact shifts obtained from the fit to Eq (3).

	H1	H2	H3ax	H3eq	H4ax	H4eq	H5ax	H5eq
Exp	15.20	24.79	133.14	44.08	133.14	68.63	-130.44	-24.14
Calcd	15.43	26.08	137.93	52.21	133.14	71.63	-115.58	-18.69

Table S2. Exchange rate values and frequency shifts obtained from the BM multi-B1 fitting procedures for $[TbL^6]^{3+}$ and $[PrL^6]^{3+}$.

$[TbL^6]^{3+}$	Peak 1	Peak 2
**Temp. (°C)**	**k_{ex} / δ**	**k_{ex} / δ**
**10**	115±1304 Hz / -70.6 ppm	14±5759 Hz / -84.8 ppm
**15**	609 ±385 Hz / -68.5 ppm	118±1172 Hz / -81.7 ppm
**20**	747±248 Hz / -66.2 ppm	83±980 Hz / -78.8 ppm
**25**	1052±139 Hz / -64.2 ppm	736±177 Hz / -76.2 ppm
**30**	1466±223 Hz / -62.2 ppm	931±294 Hz / -73.6 ppm
**35**	2174±174 Hz / -60.2 ppm	1337±159 Hz / -71.1 ppm
**37**	2454±203 Hz / -59.5 ppm	1475±173 Hz / -70.1 ppm
**40**	3074±242 Hz / -58.3 ppm	1843±200 Hz / -68.6 ppm

$[PrL^6]^{3+}$	Peak 1	Peak 2
**Temp. (°C)**	**k_{ex} / fitted δ**	**k_{ex} / fitted δ**
**25**	614±123 Hz / -2.8 ppm	173±166 Hz /-7.9 ppm
**37**	1424±345 Hz / -2.4 ppm	1160±105 Hz / -7.2 ppm

Table S3. CEST MRI on tube phantoms with **PrL⁶** and **TbL⁶** (10 mM, 25 mM PBS, pH 7.0, RT). Comparison of CEST effects under different MT parameters.

**Sat. time / [illegible]**	**PrL (-2.7 ppm)**	**PrL (-8.6 ppm)**	**TbL (-67.9 ppm)**	**TbL (-83.4 ppm)**
**5s / 10 µT**	71 %	25 %	15 %	8 %
**5s / 5 µT**	46 %	21 %	11 %	4 %
**3s / 10 µT**	66 %	23 %	15 %	7 %

Table S4. Cartesian coordinates optimized for the $[Y(H_2O)_8]^{3+}\cdot16H_2O$ system (0 imaginary frequencies).

Center Number	Atomic Number	Coordinates (Angstroms) X	Y	Z
1	39	-0.037450	0.009737	-0.036126
2	8	-1.599457	-0.294854	1.850402
3	8	0.863504	1.320436	1.697697
4	8	-1.097957	-2.049878	-0.413374
5	8	1.124401	-1.473408	1.394758
6	8	1.536201	-1.320378	-1.403448
7	8	-1.478795	1.879513	0.023914
8	8	-0.955250	0.187117	-2.209354
9	8	1.302756	1.725014	-1.166302
10	8	-4.156576	-0.850282	1.039879
11	8	-3.664943	-2.022537	-1.337612
12	8	-3.654992	0.390591	-2.543996
13	8	-4.177253	1.561012	-0.173901
14	1	-0.988086	-2.832870	0.179692
15	1	-1.985478	-2.119830	-0.837142
16	1	-1.698352	0.559963	2.366210
17	1	-2.519916	-0.554138	1.592966
18	1	-2.465335	1.853421	0.005366
19	1	-1.191484	2.744445	-0.345088
20	1	-0.573502	-0.312252	-2.965811
21	1	-1.909219	0.345681	-2.401190
22	1	2.494980	-1.074338	-1.361604
23	1	1.501118	-2.279263	-1.107019
24	1	2.101838	-1.595051	1.448807
25	1	0.736497	-1.753387	2.253635
26	1	1.790056	1.214952	2.017010
27	1	0.590830	2.251605	1.883735
28	1	1.373811	1.673414	-2.167003
29	1	2.230820	1.842809	-0.838959
30	8	4.174332	-0.696359	-1.052765
31	8	3.876301	1.872202	-0.281017
32	8	3.526429	1.073784	2.271339
33	8	3.841224	-1.494452	1.502492
34	1	4.256853	0.279459	-0.916548

35	1	4.158325	-1.373474	0.574492
36	1	3.778647	0.121246	2.199180
37	1	3.888102	1.763557	0.702323
38	1	4.367195	-2.200941	1.898472
39	1	3.938700	1.420867	3.072710
40	1	4.435631	2.630296	-0.494166
41	1	4.846327	-0.955486	-1.696346
42	1	-4.007437	0.957386	-1.815326
43	1	-4.379740	0.768397	0.381046
44	1	-4.136875	-1.435387	0.242542
45	1	-3.783716	-1.255354	-1.949557
46	1	-4.109543	-2.780900	-1.737384
47	1	-4.084527	0.670240	-3.362355
48	1	-4.817941	2.246150	0.055979
49	1	-4.806671	-1.218904	1.651734
50	8	-0.115631	3.805772	2.114886
51	8	-0.062817	4.066059	-0.594031
52	8	0.712532	-0.976822	-3.963500
53	8	1.551740	-3.887389	-0.648039
54	8	-0.525676	-4.126222	1.218690
55	8	-1.983110	1.986606	3.183502
56	8	1.595638	1.613065	-3.805663
57	8	-0.540716	-2.359899	3.291451
58	1	0.360600	4.437521	2.668523
59	1	-0.107951	4.162925	1.198800
60	1	-0.153856	4.760558	-1.258230
61	1	0.602495	3.422896	-0.926061
62	1	-1.433751	2.705941	2.807651
63	1	-1.734308	1.943988	4.115873
64	1	-0.534604	-2.372387	4.256401
65	1	-1.108283	-1.608422	3.002395
66	1	-0.556514	-3.703971	2.106728
67	1	-1.102504	-4.899902	1.252706
68	1	0.920455	-4.038850	0.086892
69	1	2.417932	-4.109062	-0.282267
70	1	1.148515	-1.339854	-3.158311
71	1	0.660515	-1.685349	-4.616612
72	1	0.982618	2.204264	-4.261644
73	1	1.332222	0.708113	-4.082399

E(RTPSSh) = -1873.3359017 Hartree
Zero-point correction = 0.611077 (/Particle)
Thermal correction to Energy = 0.667848
Thermal correction to Enthalpy = 0.668792
Thermal correction to Gibbs Free Energy = 0.527056
Sum of electronic and zero-point Energies = -1872.724825
Sum of electronic and thermal Energies = -1872.668054
Sum of electronic and thermal Enthalpies = -1872.667110
Sum of electronic and thermal Free Energies = -1872.808846

Table S5. Cartesian coordinates optimized for the $[YL^6]^{3+}\cdot 16H_2O$ system (0 imaginary frequencies, CN 9).

Center Number	Atomic Number	Coordinates (Angstroms) X	Y	Z
1	39	0.197465	-0.050649	0.095568
2	6	2.882856	-0.593971	-1.806699
3	6	4.165956	-0.375149	-2.287971
4	1	4.372162	-0.472237	-3.351003
5	6	5.166599	-0.052509	-1.384020
6	1	6.178473	0.141579	-1.733068
7	6	4.880200	-0.032228	-0.024194
8	1	5.655695	0.150406	0.715245
9	6	3.577842	-0.275776	0.378728
10	6	3.208678	-0.495504	1.827363
11	1	3.204897	-1.584539	1.966443
12	1	3.981134	-0.084795	2.492372
13	6	1.966603	1.422131	2.659197
14	1	2.826711	1.524635	3.337349
15	1	1.075865	1.644964	3.255578
16	6	2.107753	2.413612	1.517615
17	1	2.260539	3.424548	1.925437
18	1	2.988390	2.166783	0.913697
19	6	-0.232347	3.019142	1.334898
20	1	-0.026854	4.085678	1.508894
21	1	-0.369445	2.535267	2.306684
22	6	-1.507733	2.846679	0.552716
23	6	-2.440710	3.861156	0.417854
24	1	-2.266850	4.828085	0.882780
25	6	-3.587511	3.609175	-0.324969
26	1	-4.346342	4.379766	-0.441419
27	6	-3.744029	2.375062	-0.934705
28	1	-4.615857	2.151208	-1.544357
29	6	-2.747458	1.419783	-0.773697
30	6	-2.777113	0.120750	-1.536988
31	1	-3.807975	-0.187125	-1.747376
32	1	-2.285456	0.311999	-2.500059
33	6	-1.769075	-2.073645	-1.794018
34	1	-2.650851	-2.262416	-2.416021
35	1	-1.600461	-2.988995	-1.210836
36	6	-0.573194	-1.758188	-2.680337
37	1	-0.473854	-2.534110	-3.455279
38	1	-0.719445	-0.799159	-3.190268
39	6	1.756772	-1.054606	-2.707890
40	1	1.353278	-0.205907	-3.266577
41	1	2.149003	-1.782771	-3.434842
42	6	1.239186	-0.800293	3.215463
43	1	1.670080	-0.617072	4.211286
44	1	1.361366	-1.862262	2.974169
45	6	-0.256526	-0.529119	3.219933
46	6	1.185671	3.100705	-0.629423
47	1	2.244609	2.978503	-0.892817
48	1	0.983320	4.177139	-0.539351

49	6	0.417480	2.477094	-1.784271
50	6	-2.776725	-1.408955	0.351424
51	1	-2.933304	-0.548264	1.014615
52	1	-2.151550	-2.121632	0.905602
53	6	-4.134670	-2.070244	0.082518
54	6	1.099722	-2.963259	-1.385728
55	1	2.194190	-2.995545	-1.309971
56	1	0.798630	-3.770839	-2.068536
57	6	0.593042	-3.229590	0.019269
58	7	2.592537	-0.482690	-0.504447
59	7	1.869568	0.021376	2.173355
60	7	0.915320	2.384132	0.633826
61	7	-1.674387	1.646164	-0.014255
62	7	-2.040163	-0.959681	-0.843707
63	7	0.673623	-1.645942	-1.892733
64	7	-0.946905	-0.879824	4.285830
65	1	-1.951870	-0.753681	4.295607
66	1	-0.501117	-1.277492	5.102231
67	7	-0.105607	3.267447	-2.697521
68	1	-0.566888	2.862243	-3.504107
69	1	-0.032814	4.274687	-2.637398
70	7	-4.773739	-2.458942	1.200534
71	1	-5.675756	-2.907411	1.122138
72	1	-4.381489	-2.318571	2.119962
73	7	0.540685	-4.479462	0.432574
74	1	0.259884	-4.678618	1.385435
75	1	0.789737	-5.254131	-0.168684
76	8	-0.794200	-0.006852	2.219575
77	8	0.331434	1.231580	-1.852049
78	8	-4.588958	-2.231192	-1.040955
79	8	0.269844	-2.275554	0.761263

E(RTPSSh) = -1899.8496621 Hartree
Zero-point correction = 0.666079
Thermal correction to Energy = 0.702879
Thermal correction to Enthalpy = 0.703824
Thermal correction to Gibbs Free Energy = 0.601334
Sum of electronic and zero-point Energies = -1899.183583
Sum of electronic and thermal Energies = -1899.146783
Sum of electronic and thermal Enthalpies = -1899.145838
Sum of electronic and thermal Free Energies = -1899.248329

Table S6. Cartesian coordinates optimized for the $[YL^6]^{3+}\cdot 16H_2O$ system (0 imaginary frequencies, CN 10).

Center Number	Atomic Number	Coordinates (Angstroms) X	Y	Z
1	39	-0.001189	-0.000018	0.000107
2	6	3.263175	-0.113128	1.144733
3	6	4.650488	-0.134473	1.185182
4	1	5.162269	-0.247847	2.137611
5	6	5.354948	0.000794	-0.000216

6	1	6.442712	0.000796	-0.000252
7	6	4.650397	0.136044	-1.185535
8	1	5.162080	0.249392	-2.138022
9	6	3.263061	0.114688	-1.144959
10	6	2.469098	0.168112	-2.424580
11	1	3.048134	0.687313	-3.204604
12	1	2.313169	-0.865039	-2.758092
13	6	0.317701	0.542734	-3.446371
14	1	0.928849	0.634619	-4.357272
15	1	-0.457154	1.313562	-3.508364
16	6	-0.322600	-0.827334	-3.390517
17	1	-0.935578	-0.991648	-4.289893
18	1	0.451331	-1.601527	-3.392889
19	6	-2.472777	-0.372816	-2.401579
20	1	-3.051341	-0.955486	-3.135920
21	1	-2.317526	0.629067	-2.820133
22	6	-3.266556	-0.211981	-1.130930
23	6	-4.653906	-0.236189	-1.169626
24	1	-5.165416	-0.429384	-2.109277
25	6	-5.358498	-0.000707	-0.000102
26	1	-6.446258	-0.000657	-0.000139
27	6	-4.653957	0.234722	1.169483
28	1	-5.165518	0.427990	2.109092
29	6	-3.266619	0.210410	1.130878
30	6	-2.472863	0.371298	2.401535
31	1	-3.051707	0.953414	3.136084
32	1	-2.316958	-0.630614	2.819770
33	6	-0.322952	0.827154	3.390525
34	1	-0.936040	0.991087	4.289891
35	1	0.450513	1.601814	3.392909
36	6	0.318173	-0.542524	3.446365
37	1	0.929488	-0.633998	4.357196
38	1	-0.456200	-1.313822	3.508487
39	6	2.469269	-0.166651	2.424381
40	1	3.048584	-0.685371	3.204515
41	1	2.312727	0.866481	2.757666
42	6	1.278032	2.244970	-2.063258
43	1	1.626565	2.747569	-2.979362
44	1	1.984436	2.461927	-1.250645
45	6	-0.086518	2.767299	-1.638143
46	6	-1.276588	-2.411142	-1.868086
47	1	-1.629393	-2.989781	-2.736268
48	1	-1.977235	-2.561319	-1.035537
49	6	0.092172	-2.892610	-1.409601
50	6	-1.277925	2.410437	1.868184
51	1	-1.630990	2.988791	2.736447
52	1	-1.978716	2.560273	1.035706
53	6	0.090541	2.892742	1.409662
54	6	1.279407	-2.244262	2.063313
55	1	1.628195	-2.746542	2.979493
56	1	1.985978	-2.460896	1.250770
57	6	-0.084850	-2.767439	1.638283
58	7	2.581063	0.000784	-0.000099
59	7	1.151672	0.794874	-2.244920

60	7	-1.154438	-0.980859	-2.171015
61	7	-2.584480	-0.000821	-0.000012
62	7	-1.154896	0.980200	2.171017
63	7	1.152183	-0.794216	2.244839
64	7	-0.355357	4.044359	-1.835684
65	1	-1.243239	4.421843	-1.527212
66	1	0.302253	4.663479	-2.290900
67	7	0.368956	-4.178696	-1.516636
68	1	1.257456	-4.529807	-1.179788
69	1	-0.286083	-4.831120	-1.926651
70	7	0.366515	4.179016	1.516630
71	1	1.254794	4.530648	1.179744
72	1	-0.288920	4.831075	1.926594
73	7	-0.352843	-4.044682	1.835840
74	1	-1.240582	-4.422684	1.527603
75	1	0.305157	-4.663359	2.291088
76	8	-0.900813	1.985627	-1.104677
77	8	0.901998	-2.068831	-0.936168
78	8	0.900871	2.069467	0.936236
79	8	-0.899707	-1.986279	1.104923

E(RTPSSh) = -1899.873314 Hartree
Zero-point correction = 0.666925
Thermal correction to Energy = 0.702878
Thermal correction to Enthalpy = 0.703822
Thermal correction to Gibbs Free Energy = 0.604753
Sum of electronic and zero-point Energies = -1899.206389
Sum of electronic and thermal Energies = -1899.170436
Sum of electronic and thermal Enthalpies = -1899.169492
Sum of electronic and thermal Free Energies = -1899.268561

Table S7. Old-DKH-TZVPP basis set used for ^{89}Y NMR shielding tensor calculations.

```
# Basis set for element : H
 NewGTO H
 S 3
   1      34.0613410000      0.0254393168
   2       5.1235746000      0.1900859340
   3       1.1646626000      0.8524411390
 S 1
   1       0.3272304100      1.0000000000
 S 1
   1       0.1030724100      1.0000000000
 P 1
   1       1.4070000000      1.0000000000
 P 1
   1       0.3880000000      1.0000000000
 D 1
   1       1.0570000000      1.0000000000
  end;

 # Basis set for element : C
 NewGTO C
 S 6
   1   13575.3496820000      0.0008573334
   2    2035.2333680000      0.0050294039
   3     463.2256235900      0.0248779742
   4     131.2001959800      0.0983704073
   5      42.8530158900      0.3024367521
```

```
  6      15.5841857700      0.6609154327
S 1
  1       6.2067138500      1.0000000000
S 1
  1       2.5764896500      1.0000000000
S 1
  1       0.5769633900      1.0000000000
S 1
  1       0.2297283100     -1.0000000000
S 1
  1       0.0951644400      1.0000000000
P 4
  1      34.6972322400      0.0113880905
  2       7.9582622800      0.0762657660
  3       2.3780826900      0.3018857524
  4       0.8143320800      0.7278282671
P 1
  1       0.2888754700      1.0000000000
P 1
  1       0.1005682400      1.0000000000
D 1
  1       1.0970000000      1.0000000000
D 1
  1       0.3180000000      1.0000000000
F 1
  1       0.7610000000      1.0000000000
 end;

# Basis set for element : N
NewGTO N
S 6
  1   19730.8006470000      0.0009114008
  2    2957.8958745000      0.0050013971
  3     673.2213359500      0.0242730018
  4     190.6824949400      0.0959003098
  5      62.2954419000      0.2977871694
  6      22.6541611800      0.6674050439
S 1
  1       8.9791477400      1.0000000000
S 1
  1       3.6863002400      1.0000000000
S 1
  1       0.8466007700      1.0000000000
S 1
  1       0.3364713400     -1.0000000000
S 1
  1       0.1364765400      1.0000000000
P 4
  1      49.2003805100      0.0115400428
  2      11.3467905400      0.0785554359
  3       3.4273972400      0.3082057727
  4       1.1785525100      0.7201701296
P 1
  1       0.4164220500      1.0000000000
P 1
  1       0.1426082600      1.0000000000
D 1
  1       1.6540000000      1.0000000000
D 1
  1       0.4690000000      1.0000000000
F 1
  1       1.0930000000      1.0000000000
 end;

# Basis set for element : O
NewGTO O
S 6
```

```
  1   27032.3826310000      0.0009746923
  2    4052.3871392000      0.0050399649
  3     922.3272271000      0.0239325547
  4     261.2407098900      0.0942631630
  5      85.3546413500      0.2945000522
  6      31.0350352400      0.6718715898
S 1
  1      12.2608607300      1.0000000000
S 1
  1       4.9987076000      1.0000000000
S 1
  1       1.1703108200      1.0000000000
S 1
  1       0.4647474100     -1.0000000000
S 1
  1       0.1850453600      1.0000000000
P 4
  1      63.2749548000      0.0121466150
  2      14.6270493800      0.0831991531
  3       4.4501223500      0.3200101266
  4       1.5275799600      0.7069521347
P 1
  1       0.5293511800      1.0000000000
P 1
  1       0.1747842100      1.0000000000
D 1
  1       2.3140000000      1.0000000000
D 1
  1       0.6450000000      1.0000000000
F 1
  1       1.4280000000      1.0000000000
 end;

# Basis set for element :  Y
NewGTO  Y
S 8
  1  717737.9602800000      0.0019810031
  2  107596.4578400000      0.0060591225
  3   24488.2634280000      0.0182889029
  4    6931.6700144000      0.0527086200
  5    2257.4665620000      0.1436542462
  6     812.8691690300      0.3009064455
  7     316.5999928800      0.4178466650
  8     125.5492929600      0.2269131392
S 1
  1     751.4984112500      1.0000000000
S 1
  1     234.3066045200      1.0000000000
S 1
  1      40.3176505190      1.0000000000
S 1
  1      18.0009675760     -1.0000000000
S 1
  1      27.0952718610      1.0000000000
S 1
  1       5.2342184548      1.0000000000
S 1
  1       2.8442291319     -1.0000000000
S 1
  1       0.9663739281      1.0000000000
S 1
  1       0.4085983974      1.0000000000
S 1
  1       0.0632792001      1.0000000000
S 1
  1       0.0270398172     -1.0000000000
P 6
```

```
  1    3967.2077466000      0.0042155007
  2     939.9859310100      0.0246665561
  3     302.5806689200      0.1067998731
  4     113.6984414000      0.2965532118
  5      45.9367456410      0.4691445772
  6      18.8834198620      0.2823562219
P 1
  1      72.9895552650      1.0000000000
P 1
  1      11.6590726880     -1.0000000000
P 1
  1       5.3878444763      1.0000000000
P 1
  1       2.6483667788     -1.0000000000
P 1
  1       1.1871137415      1.0000000000
P 1
  1       0.5867465873     -1.0000000000
P 1
  1       0.2639562403      1.0000000000
P 1
  1       0.0650000000      1.0000000000
D 5
  1     362.2315432800      0.0051323732
  2     108.6051121400      0.0384876966
  3      41.0258584510      0.1573636380
  4      17.2025247160      0.3891804782
  5       7.4571859455      0.5705883846
D 1
  1       3.2219436625      1.0000000000
D 1
  1       1.2963782099      1.0000000000
D 1
  1       0.4230029994      1.0000000000
D 1
  1       0.1219226798     -1.0000000000
F 1
  1       0.2651500000      1.0000000000
 end;
```
